# Taming Lattice Strain via Buried Interface Engineering for Reverse-Bias Resilient Perovskite Solar Cells

**DOI:** 10.1007/s40820-026-02244-2

**Published:** 2026-05-27

**Authors:** Niqian Du, Shanshan Du, Yaru Du, Xiaobo Zhang, Xiaoyi Hou, Chi Feng, Rongdong Xiang, Xin Wu, Heping Fu, Zhiyong Liu, Tingwei He, Kaikai Liu

**Affiliations:** 1 Henan Key Laboratory of Advanced Semiconductor and Functional Device Integration, School of Physic, Henan Normal University, Xinxiang, 45007 People’s Republic of China; 2 Henan Key Laboratory of Optoelectronic Energy Storage Material and Application, School of Physics and Engineering, Henan University of Science and Technology, Luoyang, 471023 People’s Republic of China; 3 Department of Chemistry, City University of Hong Kong, Kowloon, 999077 Hong Kong People’s Republic of China; 4 College of Physics Science and Technology, Hebei University, Baoding, 071002 People’s Republic of China

**Keywords:** Buried HTL/perovskite interface, Lattice strain, Reverse-bias, Stability, Perovskite solar cells

## Abstract

Inverted perovskite solar cells have achieved exceptional efficiencies, yet their operational stability, particularly under reverse-bias stress, remains a critical challenge. This instability is fundamentally driven by lattice strain, which lowers ion migration barriers and promotes defect formation. Here, we identify the buried hole-transport-layer/perovskite interface as the principal site of strain accumulation. By incorporating 3-fluorothiophene-2-carboxylic acid (3F-2TC) at this buried HTL/perovskite interface, we directly engineer the initial perovskite crystallization template. This buried interface engineering strategy effectively alleviates intrinsic lattice strain, as unambiguously confirmed by grazing-incidence X-ray diffraction analysis. Crucially, we utilize reverse-bias stress as a diagnostic probe to decouple strain relaxation from mere defect passivation, revealing that a low-strain lattice constitutes the primary defense against bias-induced degradation. Consequently, the champion devices achieve a high power conversion efficiency (PCE) of 26.10% and markedly enhanced stability, retaining 91.58% of their initial PCE after 200 h under − 1.0 V reverse bias. This work thereby establishes the buried interface engineering for strain modulation as a generalizable design principle toward efficient and operationally resilient perovskite photovoltaics.

**Supplementary Information:**

The online version contains supplementary material available at 10.1007/s40820-026-02244-2.

## Highlights

- Modulating perovskite lattice strain, beyond merely managing ion migration or passivating defects, is pivotal for device efficiency and stability.
- Buried interface engineering simultaneously optimizes the hole-transport-layer, templates film growth and passivates defects, thereby mitigating perovskite lattice strain.
- Inverted perovskite solar cells achieve a power conversion efficiency of 26.10% with exceptional stability under reverse-bias, thermal, and operational stresses.

**Supplementary Information:**

The online version contains supplementary material available at 10.1007/s40820-026-02244-2.

## Graphical abstract

**Figure:**
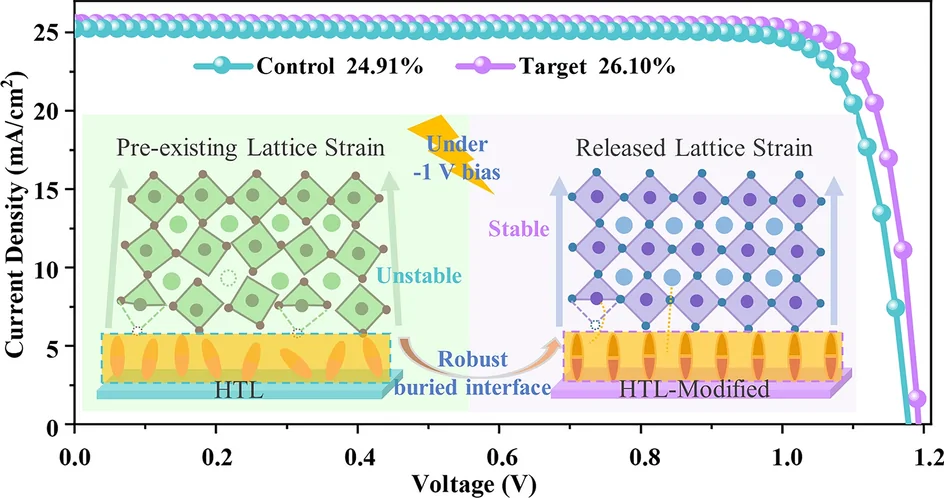

## Introduction

Inverted (p-i-n) perovskite solar cells (PSCs) have attracted considerable interest owing to their remarkable power conversion efficiencies (PCE), facile solution processability, and broad substrate compatibility [1–5]. Notably, certified PCE for these PSCs have recently surpassed 27%, closely approaching the theoretical Shockley–Queisser limit [6]. Despite these advances, the operational stability of inverted PSCs remains a formidable bottleneck hindering their real-world deployment. While mitigation strategies for extrinsic factors such as moisture, oxygen, and light have seen considerable progress [7], intrinsic degradation mechanisms originating within the perovskite bulk itself remain less elucidated. For instance, under reverse bias, a condition commonly arising in photovoltaic device when partial shading forces a shaded cell into reverse bias, triggering the hot-spot effect that causes localized overheating and accelerated degradation, devices can undergo rapid performance degradation, representing a major obstacle to commercialization [8, 9]. Such degradation is primarily driven by extensive ion migration (e.g., I^−^ anions) or defect formation (e.g., undercoordinated Pb^2+^). Crucially, both processes are intimately linked to perovskite lattice strain, which concomitantly reduces the activation barrier for ion migration and creates favorable sites for defect nucleation [10, 11]. Consequently, emerging insights reconceptualize perovskite lattice strain not as a passive byproduct, but as a fundamental driver of intrinsic instability [12]. Accordingly, developing strategies to address lattice strain are regarded as essential to achieving transformative gains in operational stability, moving beyond the conventional paradigm of solely suppressing ion migration or passivating defects.

To date, most research efforts to improve operational stability have focused on modifying either the top surface or the bulk of the perovskite layer. This is exemplified by strategies such as surface functionalization [13] and bulk additive engineering [14]. While demonstrably effective, such strategies predominantly mitigate the symptomatic consequences of strain, rather than tackling its root cause—the mismatched or defective nucleation template at the buried HTL/perovskite interface. In contrast, the buried HTL/perovskite interface, serving as a critical nucleation substrate, represents a more powerful yet underexplored locus for preemptive strain management. This is particularly critical for self-assembled monolayer (SAM)-based inverted PSCs, where the SAM/perovskite interface dictates the the crystallization quality and thereby the intrinsic stress state within the perovskite lattice [15, 16]. We thus propose that deliberate engineering at this critical point, a strategy termed “buried interface engineering,” can fundamentally tailor the film’s strain state and structural robustness. Unlike mainstream methods, this approach directly targets the root cause of instability, offering a transformative solution.

Herein, we introduce 3-fluorothiophene-2-carboxylic acid (3F-2TC) as a tailored molecular modifier at the buried HTL/perovskite interface. This intervention effectively modulates the lattice strain within the perovskite films, as confirmed through detailed structural analyses. Critically, to unambiguously assign the enhanced stability to strain relaxation rather than mere defect passivation, we devised a pivotal experiment using reverse-bias stress testing. This approach selectively accelerates strain-mediated degradation, thereby serving as a discriminative tool to confirm the role of a robust perovskite lattice. We show that 3F-2TC incorporation not only fosters a uniform, pinhole-free HTL via hydrogen bonding, but also dramatically reduces defect density at the buried THL/perovskite interface through chemical interactions with perovskite, thereby guiding the formation of high-quality, low-strain perovskite films. Consequently, the target PSCs achieve a champion PCE of 26.10% alongside markedly improved stability across harsh conditions, retaining 91.58% of their initial PCE after 200 h under − 1.0 V bias, 80.81% after 500 h at 85 °C and 90.43% illumination. This work establishes targeted molecular design at the buried HTL/perovskite interface as a foundational strategy for perovskite photovoltaics, where such design effectively regulates lattice strain to achieve both high efficiency and durability.

## Experimental Section

### Materials

CsI, MABr, MACl, PbBr_2_, and PDADI were purchased from Xian Polymer Light Technology Corp. FAI and PEABr was purchased from Great Cell Solar Materials. PbI_2_, MeO-2PACz, and Me-4PACz were purchased from Tokyo Chemical Industry (TCI). NiO_*x*_ was purchased from Advanced Election Technology Co., Ltd. MeO-4PACz was purchased from Beijing Lvxing Xiaolvren Technology. Dimethylformamide (DMF), dimethyl sulfoxide (DMSO), chlorobenzene, ethanol, and isopropanol (IPA) were purchased from Sigma-Aldrich. For the evaporation materials, BCP was purchased from Advanced Election Technology Co., Ltd. ITO substrates (2 × 2 cm^2^) were purchased from Advanced Election Technology Co., Ltd. 3-fluorothiophene-2-carboxylic acid was purchased from Aladdin. All chemicals were used directly without further purification.

### Device Fabrication

#### Preparation of Perovskite Solutions

(1) Regular-bandgap (~ 1.54 eV): 1.5 M Cs_0.05_(MA_0.05_FA_0.95_)_0.95_Pb(I_0.95_Br_0.05_)_3_ of perovskite precursor solution was prepared in a N_2_-filled glovebox. Specifically, 19.48 mg CsI, 8 mg MACl, 8.13 mg MABr, 232.95 mg FAI, 677 mg PbI_2_, and 28.07 mg PbBr_2_ were dissolved in 1 mL of a solvent mixture composed of DMF and DMSO at a volume ratio of 4:1. Then, the prepared perovskite solution was sealed with parafilm in the glovebox and transferred to the oscillator for 4–6 h. Lastly, the solution was filtered with a 0.22-μm polytetrafluoroethylene (PTFE) filter in the glovebox before spin coating.

(2) Wide-bandgap (1.68 eV): 1.3 M Cs_0.05_MA_0.15_FA_0.8_Pb(I_0.76_Br_0.24_)_3_ of perovskite precursor solution was prepared by dissolving 16.9 mg CsI, 21.84 mg MABr, 178.88 mg FAI, 143.13 mg PbBr_2_, and 422.11 mg PbI_2_ in 1 mL of abovementioned DMF/DMSO mixed solvent.

(3) Wide-bandgap (1.78 eV): 1 M Cs_0.2_FA_0.8_Pb(I_0.6_Br_0.4_)_3_ of perovskite precursor solution was prepared by dissolving 32.7 mg CsI, 86.9 mg FAI, 42 mg FABr, 136.9 mg PbBr_2_, and 312.4 mg PbI_2_ were dissolved in 1 mL of abovementioned DMF/DMSO mixed solvent.

#### Solar Cell Fabrication

(1) Regular-bandgap perovskite solar cells (~ 1.54 eV-based PSCs): Perovskite solar cells were prepared on the pre-patterned ITO glass. The substrates were cleaned by ultrasound using detergent, deionized water, acetone, and isopropanol for 15 min each. Then, the ITO substrates were dried using a N_2_ gun, followed by plasma treatment for 5 min. Subsequently, 0.3 mg mL^−1^ MeO-4PACz in ethanol was spin-coated on the substrate at 3000 rpm for 30 s in a nitrogen glovebox, followed by annealing at 100 ℃ for 10 min. For the target device, the modified SAM solutions are prepared by mixing MeO-4PACz (0.3 mg mL^−1^ in ethanol) with 3-fluorothiophene-2-carboxylic acid (1 mg mL^−1^ in ethanol) in different volume ratios, then stirred for 30 min before deposition. Owing to its methoxy (-OCH₃) groups, MeO-4PACz exhibits good wettability on bare ITO, enabling direct processing without a NiO_*x*_ interlayer. The fabrication process of MeO-2PACz is same to the MeO-4PACz. Afterward, the filtered perovskite precursor solution was spin-coated onto the substrate using the one-step method. The substrate was spinning at 1000 rpm for 10 s (acceleration is 1000) and 5000 rpm for 30 s (acceleration is 1000). When the countdown reached 10 s, 120 μL of chlorobenzene, serving as the antisolvent, was dropped onto the spinning substrates. After that, the substrate was transferred to a thermostatic heater and annealed at 100 ℃ for 30 min. Then, 2 mg mL^−1^ PEABr in IPA was spin-coated on the perovskite film at 3000 rpm for 30 s. Next, 20 mg mL^−1^ PCBM in CB was spin-coated on the substrate at 2000 rpm for 30 s. Afterward, samples were transferred to a thermal evaporator where 6 nm of BCP and 100 nm of Ag were sequentially deposited. The active area of the fabricated device was 0.071 cm^2^.

(2) Wide-bandgap perovskite solar cells (WBG PSCs): ITO substrates were treated following the same procedure as that used for ~ 1.54 eV-based PSCs. NiO_*x*_ (10 mg mL^−1^ in H_2_O) was spin-coated onto the ITO substrate at 4000 rpm for 20 s, followed by annealing in ambient air at 100 °C for 10 min. Subsequently, Me-4PACz (0.5 mg/mL in ethanol) was deposited onto the NiO_*x*_ substrate at 3000 rpm for 30 s and annealed in a nitrogen glovebox at 100 °C for 10 min. For the perovskite layer deposition (1.68 eV), the substrate was spun at 1000 rpm for 8 s, followed by 5000 rpm for 30 s and 200 μL of chlorobenzene, serving as the antisolvent, was dropped within the last 5 s onto the spinning substrates. For the perovskite (1.78 eV), a similar spin-coating procedure was applied, with a continuous spin at 5000 rpm for 50 s, and 400 μL of ethylacetate was dropped at 30 s during the spinning process. The as-prepared perovskite films were then annealed at 100 °C for 20 min. Thereafter, a solution of 1,3-diaminopropane dihydroiodide (PDADI) in isopropanol (1 mg mL^−1^) was spin-coated onto the perovskite layer at 3000 rpm for 30 s and annealed at 100 °C for 5 min. The electron transport layer and electrodes were fabricated following the same procedures as those used for the ~ 1.54 eV-based PSCs.

### Film Characterization

X-ray diffraction (XRD) was measured with an X-ray diffractometer (Bruker D-8 Discovery). Grazing-incidence X-ray diffraction (GIXRD) measurements were performed on a Malvern Panalytical X’Pert Powder diffractometer (Netherlands) using Cu Kα radiation (*λ* = 1.5418 Å). The incident angle of X-ray was 10°, and the samples were prepared using the mechanical exfoliation method to expose the buried HTL/perovskite interface, enabling direct probing of the lattice strain at this interface. The morphology of the perovskite films was characterized by a field-emission scanning electron microscope (SEM, ZEISS SUPRA40). X-ray photoelectron spectroscopy (XPS) was measured with ESCALAB 250Xi (Thermo Fisher Scientific). Atomic force microscopy (AFM) and Kelvin probe force microscopy (KPFM) were measured using the MultiMode 8 atomic force microscope (Bruker Corporation, USA) instrument. Fourier-transform infrared (FTIR) spectroscopy was conducted with the Bruker Vertex-80. Ultraviolet photoelectron spectroscopy (UPS) was acquired by Thermo Scientific ESCA Lab 250Xi. The absorption and transmission spectra were measured on an ultraviolet–visible spectrophotometer (UV-3600, Shimadzu). The photoluminescence (PL) mapping, steady-state PL and the time-resolved photoluminescence (TRPL) were recorded with a confocal microscope (FastFLIM Q2) using a pulsed diode laser (PDL-800 LDH-P-C-470) at 405 nm for excitation. Dynamic light scattering (DLS) measurements were carried out on a Zetasizer Pro (Malvern, UK). Femtosecond transient absorption spectroscopy (fs-TA) was collected on the Ultrafast System (Helios pump-probe system, Coherent Co.). Excitation was provided by a Ti:sapphire amplifier delivering femtosecond pulses (25 fs) centered at 800 nm, operating at 1 kHz with pulse energies reaching 4 mJ. We note that excitation fluence employed may induce hot-carrier effects; accordingly, the TA kinetics are interpreted as a qualitative comparative probe.

### Device Characterization

The *J-V* curves were measured by a B2901BL source meter and an EnliTech solar simulator AM 1.5 G 100 mW cm^−2^ with a silicon solar cell for 1-sun light-intensity calibration. The voltage values were scanned at a 0.02 V step in the range of − 0.2 to 1.2 V. To enhance the accuracy of the *J–V* measurements, we employed a mask to determine a working area of 0.053 cm^2^ for PSCs. Space-charge-limited current (SCLC) and dark current tests were performed under dark conditions to characterize defect density and leakage current, respectively. In addition, light-intensity-dependent voltage and current tests were performed under different light intensities. The ion migration activation energy (*E*_a_) was determined from temperature-dependent conductivity measurements performed in the dark using a homemade heating/cooling stage and a semiconductor parameter analyzer (KEYSIGHT B1500A). The steady power output (SPO) is obtained by performing the current–time (I–T) monitor at the maximum power point (MPP). External quantum efficiency (EQE) measurements were conducted with a QE system (Enli Tech.). Thermal admittance spectroscopy (TAS) measurements were carried out using Agilent’s thermal admittance spectroscopy system to obtain conduction–capacitance–frequency maps of the PSCs. The distribution of energy levels and defect-state density was obtained by the formula conversion. Mott–Schottky and electrochemical impedance spectroscopy (EIS) measurements were recorded by the electrochemical workstation (CHI660E). Transient photocurrent (TPC) and transient photovoltage (TPV) were measured with a PAIOS system (Fluxim AG, Switzerland).

### Reverse-Bias Treatment

Electrical characterization involved applying a reverse bias to the perovskite solar cells with a B2901BL source meter and evaluating their performance through *J–V* and TAS measurements. This was followed by systematic analysis of the structural and morphological evolution of the perovskite films using XRD and SEM.

### Theoretical Calculation

#### Density Functional Theory Calculation Methods of Adsorption Energy

The molecular geometry optimization and surface charge distribution were performed using density functional theory (DFT) with the B3LYP functional and the 6-31G* basis set, implemented in the Materials Studio DMol3 module. The optimized structure was confirmed as a local minimum by frequency analysis. First-principles calculations of crystal structural optimizations and energy-related computations were carried out using the Quickstep module of the CP2K software package [17]. Structural optimizations were performed using the PBEsol functional combined with DFT-D3 dispersion corrections to account for weak interactions, while single energy calculations employed the HSE06 hybrid functional with auxiliary density matrix methods (ADMM). The pseudopotentials were based on the TZVP-MOLOPT-GTH basis set, with a plane-wave cutoff energy of 450 Ry. The convergence criterion for the density matrix in the inner self-consistent field (SCF) loop was set to 1 × 10^–6^. The adsorption energy (*E*_ads_) is calculated using the following formula:

1 $${E}_{\mathrm{a}\mathrm{d}\mathrm{s}}={E}_{\mathrm{t}\mathrm{o}\mathrm{t}\mathrm{a}\mathrm{l}}-({E}_{\mathrm{s}\mathrm{l}\mathrm{a}\mathrm{b}}+{E}_{\mathrm{m}\mathrm{o}\mathrm{l}\mathrm{e}\mathrm{c}\mathrm{u}\mathrm{l}\mathrm{e}})$$

where ${E}_{\mathrm{t}\mathrm{o}\mathrm{t}\mathrm{a}\mathrm{l}}$ is the total energy of the relaxed adsorption system (ITO + CoSAM), ${E}_{\mathrm{s}\mathrm{l}\mathrm{a}\mathrm{b}}$ represents the energy of the optimized ITO clean surface, and ${E}_{\mathrm{m}\mathrm{o}\mathrm{l}\mathrm{e}\mathrm{c}\mathrm{u}\mathrm{l}\mathrm{e}}$ denotes the energy of the isolated molecule (MeO-4PACz or FTHCA). Multiwfn, VMD and VESTA are used to analyze and visualize electronic structure data [18–21].

#### Pb-I-Pb Bond Angle

First-principles calculations were conducted to optimize the perovskite crystal structure employing the Quickstep module within the CP2K software package. The surface model was generated by cleaving the FAPbI_3_ unit cell along the [001] crystallographic direction to achieve a PbI_2_-terminated surface. Subsequently, a 3 × 3 × 3 supercell was constructed from this configuration. To mitigate spurious periodic interactions, a vacuum layer of 30 Å was introduced in the direction perpendicular to the surface, accompanied by the application of a dipole correction along the Z-axis. The computational approach utilized the PBEsol exchange–correlation functional, enhanced with the DFT-D3 method to account for van der Waals interactions. The electron wavefunctions were expanded using a double-zeta valence polarized (DZVP) Gaussian-type MOLOPT basis set, in conjunction with a plane-wave cutoff of 400 Ry. All atomic species were represented using Goedecker–Teter–Hutter (GTH) pseudopotentials. The self-consistent field (SCF) calculations were performed using the orbital transformation (OT) method, with an energy convergence criterion set to 1.0 × 10^–6^ Hartree.

## Results and Discussion

### Correlating the Reverse-Bias Stability with Lattice Strain

**Fig. 1:**
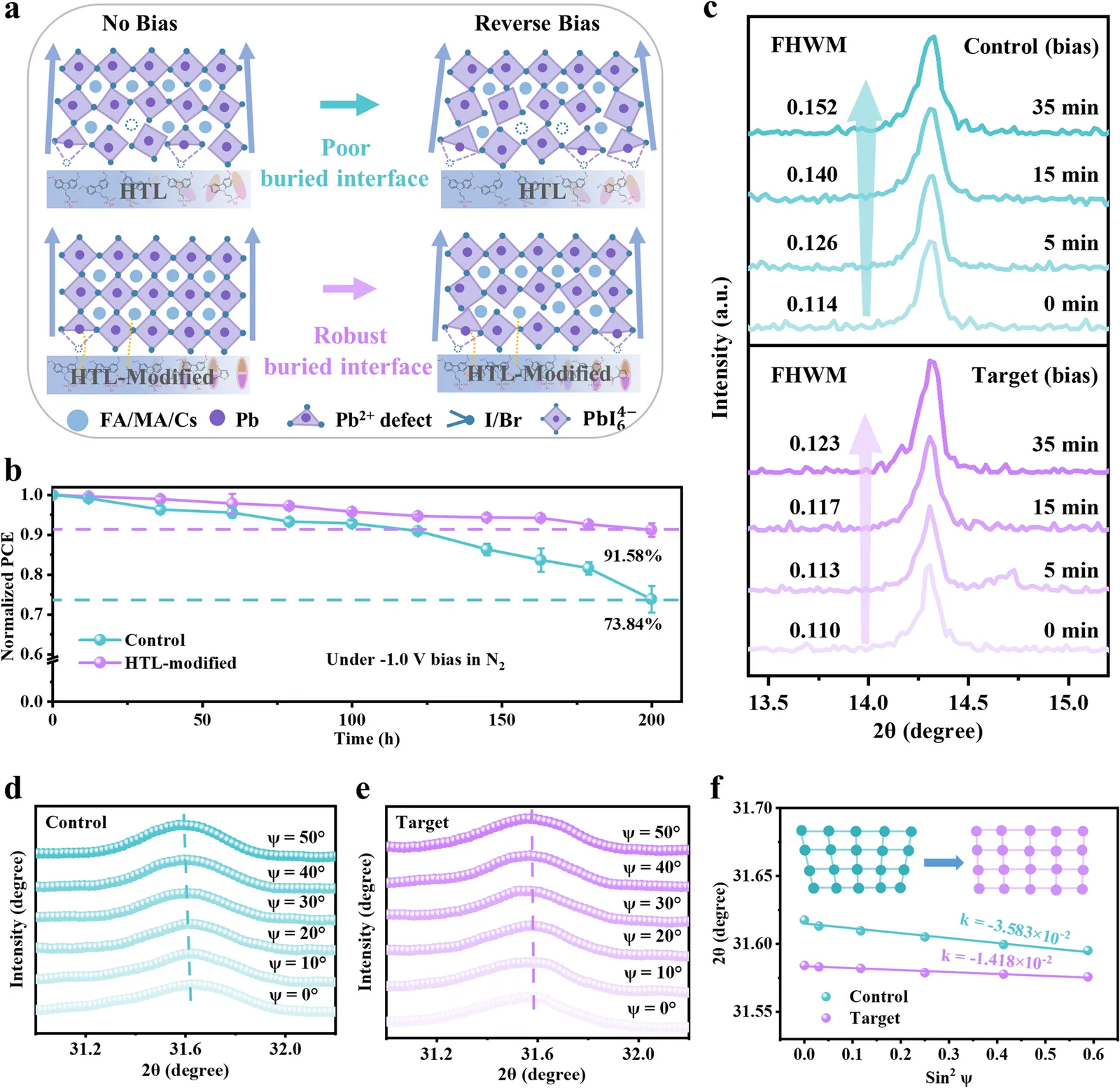
Reverse-bias treatment for perovskite films deposited on HTL or HTL-modified: **a** Schematic diagram of the lattice change. **b** PCE changes of corresponding devices. **c** XRD analysis of the (100) diffraction peak full width at half maximum (FWHM) under reverse bias. **d, e** GIXRD spectra of the control and target perovskite films. **f** Linear fittings of 2*θ*-sin^2^*ψ* obtained from the GIXRD measurement

As illustrated in Fig. 1a, the buried HTL/perovskite interface serves as a critical site for the accumulation of lattice strain, which ultimately triggers device degradation under various accelerated-aging conditions, particularly under reverse-bias stress. To elucidate the influence of buried interfacial quality on both perovskite lattice strain and device stability, we adopted reverse bias as an aggressive accelerated-aging stimulus to amplify the underlying effects and verify their correlation. Initially, we fabricated devices based on two distinct HTL configurations: a control HTL comprising only MeO-4PACz and a target HTL by incorporating 3F-2TC into MeO-4PACz; molecular structures are provided in Fig. S1. Note that systematic optimization of the blending ratio of 3F-2TC to MeO-4PACz has been conducted (Fig. S2), and unless otherwise specified, all target samples employed this optimized ratio of 10 vol% for 3F-2TC incorporation. Subsequently, we applied a reverse bias to the PSCs to assess its impact on device stability and the corresponding perovskite crystallization characteristics.

Initial screening at various bias voltages reveals comparable performance at 0 V (Fig. S3). Under the high reverse bias (Fig. S4), both the control and target devices exhibit severely degradation. At − 1.0 V, however, although the control device undergoes substantial degradation, the target device remains stable. Consequently, − 1.0 V was adopted as the stress condition for subsequent tests. Figure 1b presents reverse-bias stability testing under − 1.0 V, confirming this trend that the control device suffers significant PCE decay, while the target device retains 91.58% of its initial PCE after 200 h, demonstrating superior reverse-bias stability. More importantly, although the PCE of the target device dropped from 25.07 to 21.75 after 8 h of reverse-bias stress at − 1.0 V, it exhibited almost complete PCE self-recovery after overnight dark storage, demonstrating the resilient behavior (Fig. S5), consistent with the previous report [22]. These results demonstrate that modifying the buried HTL/perovskite interface with 3F-2TC effectively suppresses bias-induced degradation, a phenomenon attributable to its beneficial modulation of the perovskite crystal structure.

To identify the structural origin of these improvements, we conducted XRD analysis to monitor the structural evolution of perovskite films subjected to a − 1.0 V reverse bias for different times. Our analysis focused on the (100) diffraction peak, which serves as a primary indicator of the perovskite phase quality and its crystalline integrity. As shown in the magnified XRD patterns in Fig. 1c, the control sample exhibits distinct diffraction peaks corresponding to the (100) planes. With increasing reverse-bias duration, however, the full width at half maximum (FWHM) of these peaks broadened from an initial value of 0.114 to 0.152, indicating a degradation of the crystalline structure [23]. In contrast, under the same conditions, the target sample exhibited a much more moderate broaden change of the FWHM, with the value shifting from 0.110 to 0.123. This comparison reveals superior crystallization stability for the target sample, which we attribute to the favorable perovskite crystallization enabled by the improved buried HTL/perovskite interface resulting from 3F-2TC incorporation [24].

To gain deeper insight into the perovskite lattice strain, we further performed GIXRD analysis on the bottom interfaces of the control and target perovskite films, using a grazing-incidence angle of 10°, which provides a probing depth sufficient to cover the entire film from the buried interface to the top surface. Furthermore, Fig. S6 shows the schematic diagram of preparing the buried HTL/perovskite interface. As shown in Fig. 1d, e, the (210) diffraction peak for the control sample shifts from 31.617° to 31.595° with increasing the tilt angle *ψ*, while the corresponding peak for the target sample remains essentially unchanged at ~ 31.580°. The slope of the fitting curve of 2*θ*-sin^2^ *ψ* reflects the magnitude of residual stress in perovskite films. From the sin^2^ *ψ* plots (Fig. 1f), the control sample yields a slope of − 3.583 × 10^–2^, signifying the presence of considerable lattice strain [25]. By contrast, the target sample exhibits a near-zero slope of − 1.418 × 10^–2^, indicating reduced lattice distortion and released residual strain. This results in a more uniform strain distribution throughout the perovskite film, which effectively suppresses the formation of stress-induced defects and inhibits ion migration.

The ion migration activation energy (*E*_a_) of in perovskite films are measured in Fig. S7. The target device exhibits a higher *E*_a_ (0.63 eV) than the control (0.47 eV), indicating effective ion migration inhibition [26]. This is attributed to 3F-2TC releasing lattice strain, which suppresses strain-induced defects and thereby enhances device stability [27]. These above findings imply that modifying the buried HTL/perovskite interface reduces perovskite lattice strain, thereby steering the perovskite film toward lower intrinsic strain and consequently superior structural stability. It is well established that structural instability in perovskite crystals arises from defect generation and ion migration, both of which are governed by the intrinsic lattice strain [28]. Therefore, modulating the lattice strain within perovskite films is essential to rationally manage defect formation energies and elevate ion migration barriers**—**an objective attainable through strategic modification of the buried perovskite interface.

### Enhancement of Perovskite Film Quality Under Reverse Bias

**Fig. 2:**
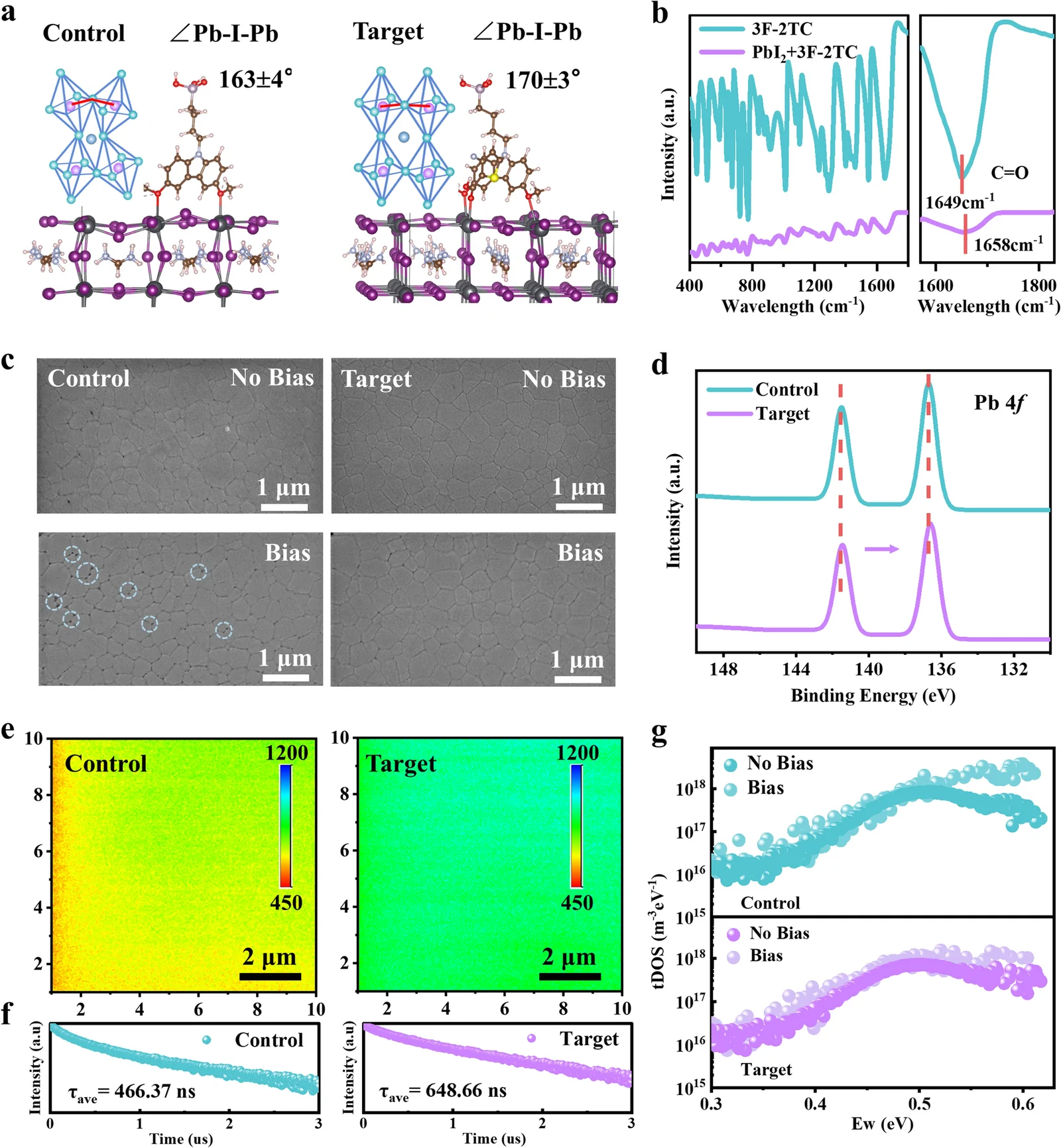
Characterizations for the buried HTL/perovskite interface of the control perovskite film and target perovskite film: **a** DFT calculation for Pb-I-Pb bond angels of the perovskite. **b** FTIR spectra of 3F-2TC and 3F-2TC/PbI_2_ mixture. **c** SEM morphology before and after reverse bias of − 1 V. **d** Pb 4*f* spectra of XPS measurements. **e** PL mapping images. **f** TRPL results. **g** tDos plots before and after reverse bias of − 1 V

The perovskite film quality, particularly of its buried interfacial quality, is critically governed by the underlying substrate. To gain theoretical insight, we employed DFT calculations to predict the effect of buried interfacial modification mediated by 3F-2TC on the perovskite lattice. As illustrated in Fig. 2a, incorporating 3F-2TC promotes Pb-I-Pb bond angles closer to the ideal 180°, facilitating a more stable perovskite lattice with reduced intrinsic strain. We attribute these changes in bond angles to the consequence of the chemical interaction between 3F-2TC and perovskite (PbI_2_, FAI), as confirmed by FTIR spectroscopy. Specifically, in the 3F-2TC/PbI_2_ mixture, the C=O stretching vibration undergoes a significant red-shift (from 1649 to 1658 cm^−1^, Fig. 2b), providing direct evidence for the passivation of uncoordinated Pb^2+^ ions. Concurrently, a measurable blue-shift (from 3337 to 3328 cm^−1^, Fig. S8) of the N–H stretching vibration in the 3F-2TC/FAI mixture suggests the formation of N–H···F hydrogen bonding, which contributes to stabilizing FA^+^ ions [29]. SEM images of the buried perovskite interface (Fig. 2c) show that the control film contains distinct voids, a typical result of solvent (DMF/DMSO) evaporation during annealing [25], and these voids become significantly more pronounced after reverse-bias treatment. In contrast, the target film exhibits a compact, uniform microstructure with large grains, and this superior morphology featuring minimal voids remains well-preserved even after reverse-bias treatment. The microstructural improvement stems from the optimized buried HTL/perovskite interface mediated by 3F-2TC, which promotes more favorable perovskite crystallization [30].

We also used various measurements to verify the improved perovskite crystallization. XPS analysis of the HTL substrate and the buried perovskite films (Figs. S9 and S10) revealed a distinct F 1*s* signal for the target sample, confirming the retention of 3F-2TC at the buried HTL/perovskite interface. Moreover, the chemical shift of the F signal indicates its active interaction with the perovskite, consistent with the proposed passivation mechanism. Critically, the Pb 4*f* signals for the target film exhibit a distinct down-shift in binding energy relative to the control (Fig. 2d), evidencing a Lewis acid–base interaction between the C=O group of 3F-2TC and undercoordinated Pb^2+^ sites [31]. This defect-passivation effect is further corroborated by a corresponding down-shift for the I 3*d* signal (Fig. S11). As shown in Fig. 2e, with more uniform and intense PL mapping, the target film shows effective defect passivation and suppressed non-radiative recombination. TRPL analysis quantitatively supports this conclusion, revealing a prolonged average carrier lifetime of 648.66 ns for the target film, compared to 466.37 ns for the control film (Fig. 2f and Table S1) [32]. Taken together, these characterizations confirm that 3F‑2TC-mediated efficient defect passivation enhances the quality of the buried HTL/perovskite interface, which, in turn, optimizes charge transfer in the device [33].

The trap densities (*N*_t_) within perovskite films were quantified via the SCLC method using hole-only devices (Fig. S12). The trap-filled limit voltage (*V*_TFL_) reflects the *N*_t_ of the perovskite film [34], which can be calculated according to Eq. S1. The target device exhibits a lower *V*_TFL_ of 0.66 V, corresponding to a reduced trap density of 3.21 × 10^15^ cm^−3^, compared to the control device (*V*_TFL_ = 0.83 V, *N*_t_ = 4.04 × 10^15^ cm^−3^). TAS result corroborates this reduction, particularly for deep-level traps (0.5 ~ 0.6 eV) associated with undercoordinated Pb^2+^ (Fig. 2g). Importantly, reverse-bias treatment drastically increases deep-level traps in the control film, while the target film exhibits minimal change. We attributed this reverse-bias resilience to the robust perovskite lattice and suppressed ion migration resulting from the superior buried perovskite interface. We further analyzed the surface and bulk morphological properties of perovskite films. The target film exhibits a reduced root-mean-square (RMS) surface roughness of 19.6 nm, compared with 22.2 nm for the control film (Fig. S13). Cross-sectional SEM images show that the target film possesses a monolithic structure with aligned vertical growth and fewer grain boundaries, in contrast to the irregular grains for the control film (Fig. S14). Furthermore, the target film demonstrates enhanced light absorption in the 500–600 nm range (Fig. S15), which facilitates more efficient photon harvesting within the perovskite films.

The above structural and chemical characterizations consistently demonstrate that 3F-2TC modification at the buried HTL/perovskite interface promotes a compact, low-defect perovskite film with substantially reduced lattice strain. Such a structurally robust interface is expected to not only suppress non-radiative recombination but also facilitate charge extraction and transport.

### Modification of Carrier Dynamics at the Buried HTL/Perovskite Interface

**Fig. 3:**
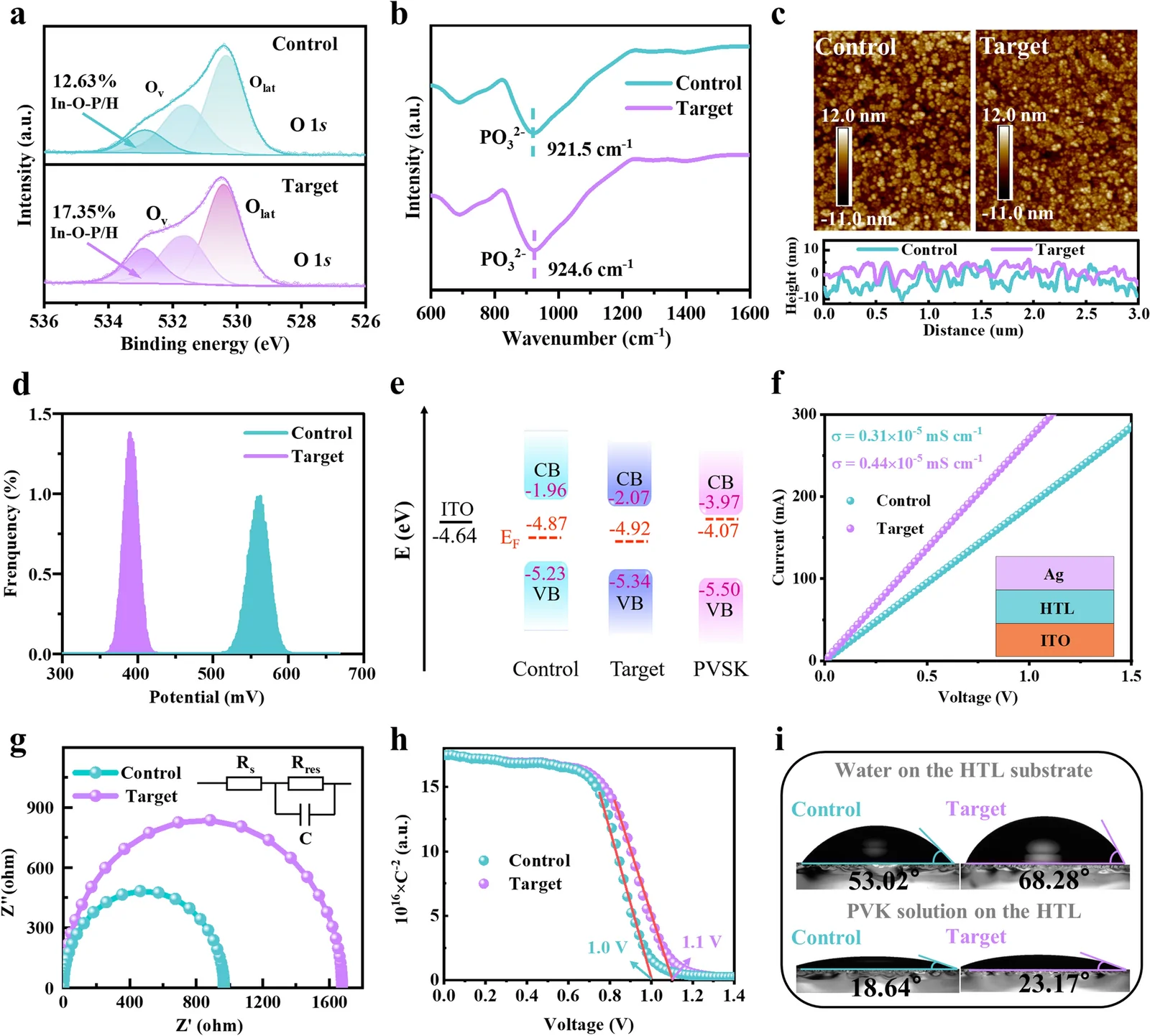
**a** O 1*s* signal of XPS spectrum of the control HTL and target HTL. **b** FTIR spectra of the control HTL and target HTL deposited on ITO substrates. **c** AFM images of the control HTL and target HTL, and corresponding height lines. **d** Surface CPD extracted from KPFM images. **e** Corresponding energy-level diagram. **f** Conductivity curves based on the ITO/HTL (control or target)/Ag structure. **g** EIS spectra (The inset is the equivalent circuit model) and **h** Mott–Schottky curves, which were recorded from the completed devices. **i** Contact angle of the control HTL and target HTL with water (top) and perovskite precursor solution (bottom)

Generally, the HTL, as the starting point for perovskite growth, directly guides the film quality of the upper perovskite layer. However, the amphiphilic nature of MeO-4PACz tends to drive its self-aggregation into micelles, resulting in insufficient anchoring to ITO and giving rise to non-uniform HTLs with pinholes, thereby degrading the integrity of the buried HTL/perovskite interface [35, 36]. In here, integrating 3F-2TC can facilitate the construction of a uniform HTL with high coverage on ITO. As seen in XPS analysis, the area ratio of the In–O–P/In–O–H component is markedly elevated in the target sample (17.35%) relative to the control (12.63%), indicating enhanced SAM coverage on the ITO surface (Fig. 3a). This enhancement is attributed to the reinforced chemical bonding between the –PO(OH)_2_ groups of MeO-4PACz and the ITO substrate [37], as evidenced by conspicuous downward shifts in the P 2*p* and In 3*d* binding energies observed for the target sample (Fig. S16a, b). Moreover, a red-shift in the PO_3_^2−^ stretching vibration of the target HTL provides additional evidence for the enhanced MeO-4PACz/ITO interfacial anchoring facilitated by 3F-2TC (Fig. 3b).

The enhanced anchoring of MeO-4PACz on the ITO substrate is conducted deeply. According to the calculations, the binding energies of MeO-4PACz dimers and tetramers are − 0.217 and − 0.379 eV, respectively (Fig. S17a). Such aggregation, arising from intermolecular hydrogen bonding, subsequently induces detrimental pinholes in the SAM layer (Fig. S17b). Dynamic light scattering (DLS) confirms that the 3F-2TC reduces the average hydrodynamic diameter of the MeO-4PACz micelles, demonstrating its inhibitory effect on micelle formation (Fig. S18). The 3F-2TC molecule, featuring the carboxyl (–COOH) and fluorine (–F) groups, suggests potential interaction sites for interaction with MeO-4PACz (Fig. S19). Consistently, the MeO-4PACz/3F-2TC heterodimer exhibits a higher binding energy of − 0.458 eV, strongly suggesting the existence of these potential interactions, likely through hydrogen bonding (Fig. S20). FTIR results provide direct evidence for these potential interactions, as indicated by characteristic peak shifts, thereby confirming the formation of hydrogen bondings (Fig. S21) [38–40]. These interactions suppress the self-aggregation of MeO-4PACz and guide their ordered assembly on ITO, as corroborated by the increased adsorption energy of MeO-4PACz on ITO (from − 9.84 to − 11.04 eV, Fig. S22). Consequently, incorporating 3F-2TC facilitates the formation of a denser and uniform SAM-based HTL [41].

The target HTL also demonstrates significant improvements in both morphology and physical properties. Specifically, compared with the control HTL, the target HTL exhibits not only a smoother surface with root-mean-square roughness (Rq) of 2.80 nm (Fig. 3c), which facilitates intimate contact with the perovskite film, thereby reducing trap state density and minimizing leakage current [42], but also exhibits a lower surface potential (Figs. 3d and S23). This suggests a downward shift of the Fermi level (*E*_f_), which facilitates optimized the interfacial energy-level alignment [43, 44]. UPS measurements corroborate this energy-level optimization (Fig. S24), showing that it reduces interfacial energy barriers and enhances charge extraction/transport at the buried HTL/perovskite interface, as schematically summarized in Fig. 3e. Furthermore, current–voltage (I–V) analysis of the ITO/HTL/Ag structure (Fig. 3f) confirms the enhanced conductivity of the target HTL [45, 46]. Moreover, analysis of the local integral density difference at the ITO/target HTL interface (Fig. S25) reveals pronounced oscillatory behavior with increased amplitude, suggesting enhanced interfacial charge transfer [47].

To investigate the impact of 3F‑2TC on interfacial carrier dynamics, we performed fs-TA on perovskite films deposited on HTL/ITO substrate without and with 3F‑2TC modification. As shown in the 2D fs-TA color maps (Fig. S26a, b), both films exhibit ground-state bleaching (GSB) peaks at ~ 766 nm, characteristic of carrier-induced band-edge filling. Following normalization by the steady-state absorbance at 766 nm, the target sample exhibits a relatively accelerated initial decay component and a moderately shortened carrier lifetime relative to the control (Fig. S26c, Table S2). These results indicate that 3F‑2TC modification facilitates photogenerated hole extraction from the perovskite to the HTL. Furthermore, PL quenching measurements were conducted to evaluate interfacial hole extraction (Fig. S27). Compared with the control sample, the target film exhibits a discernible decrease in PL intensity, indicating more efficient hole extraction at the 3F-2TC-modified perovskite/HTL interface. EIS reveals an increased recombination resistance (Rrec, from 963.05 Ω of the control to 1670.69 Ω) for the target device (Fig. 3g, Table S3), confirming the improved interfacial quality, which helps to raise the activation energy for ion migration and block the primary pathways for ion leakage [48]. Mott–Schottky analysis (Fig. 3h, Eq. S3) reveals a higher built-in potential (*V*_bi_) for the target device (1.1 vs. 1.0 V), implying a stronger driving force for charge transport, and reducing charge accumulation [49]. Consistently, this enhanced Rrec and *V*_bi_ can be attributed to the reduced lattice strain enabled by 3F-2TC modification at the buried HTL/perovskite interface.

Furthermore, as shown in Fig. 3i, the target HTL demonstrates larger contact angles for both water (68.28° vs. 53.02°) and the perovskite precursor solution (23.17° vs. 18.64°), indicating enhanced surface hydrophobicity [50, 51]. Such enhanced hydrophobicity not only effectively mitigates the interfacial stress during perovskite deposition, but also suppresses heterogeneous nucleation and promotes a compact, void-free perovskite morphology (as evidenced by the SEM images). In addition, it improves the HTL’s chemical stability and maintains the structural integrity to prevent pinhole formation. Furthermore, the target HTL film shows modestly higher light transmittance in the 400–500 nm range (Fig. S28), which may enhance light harvesting in perovskite films. Taken together, incorporating 3F-2TC effectively optimizes interfacial quality and enhances charge transport and extraction at the buried HTL/perovskite interface.

### Photovoltaic Performance and Stability of PSCs

**Fig. 4:**
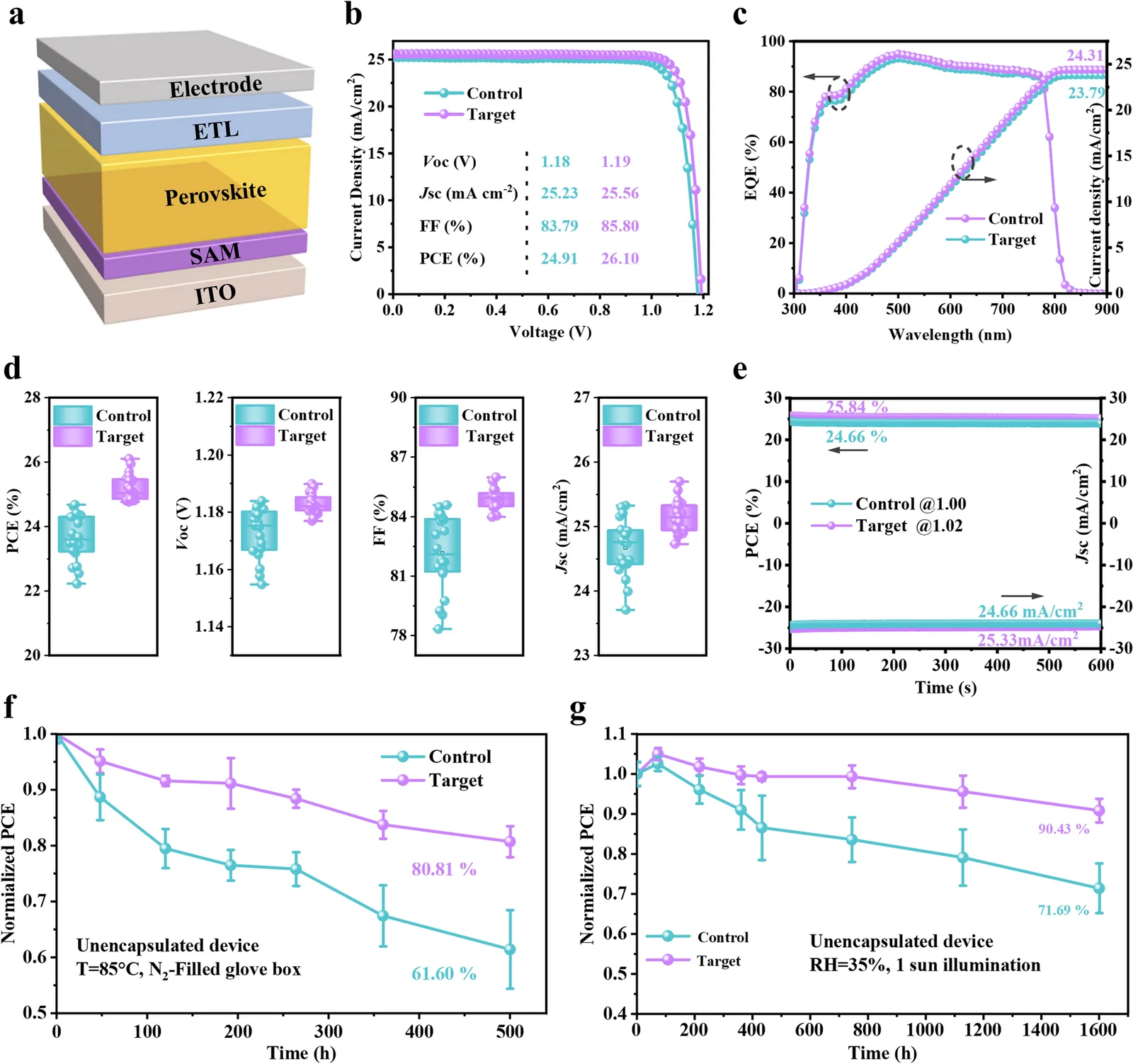
Photovoltaic performances for the control and target devices: **a** schematic of the device structure. **b** *J–V* curves under the reverse scan. **c** Corresponding EQE spectra. **d** Distribution statistics of PCE, *V*_OC_, FF, and *J*sc. **e** Steady-state output (SPO) at the maximum power point. **f** Thermal stability of unencapsulated devices in N_2_ (Error bars represent the standard deviation from four devices). **g** PCE evolution of unencapsulated devices under continuous illumination with 1-sun intensity at the air atmosphere

To evaluate the photovoltaic performance, planar devices with the architecture of ITO/SAM/perovskite/ETL/Ag were fabricated (Fig. 4a). The champion device delivers a high PCE of 26.10% with an open-circuit voltage (*V*oc) of 1.19 V, a fill factor (FF) of 85.80%, and a short-circuit current (*J*sc) of 25.56 mA cm^−2^ (Fig. 4b). By contrast, the control device exhibits a lower PCE of 24.91% (*V*oc = 1.18 V, FF = 83.79%, and *J*sc = 25.23 mA cm^−2^). The integrated current density from the external quantum efficiency (EQE) spectrum (Fig. 4c) shows good agreement with the *J–V* measurement, confirming the reliability of the photocurrent. Notably, the target device displays a reduced hysteresis index (1.9% vs. 3.7%, Fig. S29, Eq. S4), which can be ascribed to the optimized energy-level alignment that suppresses charge accumulation at the buried HTL/perovskite interface. Statistical analysis of 20 devices (Fig. 4d) confirms the superior and reproducible performance of the target devices across all key photovoltaic parameters (PCE, *V*_OC_, FF and* J*_SC_).

The enhancements in *V*oc and FF primarily stem from the uniform and dense HTL, combined with effective defect passivation at the buried HTL/perovskite interface, both of which are facilitated by 3F-2TC incorporation. Meanwhile, the increased *J*sc arises from enhanced light harvesting within the perovskite layer. We also extended this buried interface engineering strategy to other systems. As shown in Figs. S30 and S31, 3F-2TC-modified PSCs consistently enhanced the device performance across different SAM architectures and perovskite compositions, demonstrating its universality as the buried interface modulator. TPV measurements demonstrate an extended charge recombination lifetime in the target device (0.21 vs. 0.17 s for the control, Fig. S32, Eqs. S5 and S6). Correspondingly, light-intensity-dependent *V*oc analysis yields a lower ideality factor (1.13 vs. 1.46 K_B_T/q, Fig. S33, Eq. S7), indicating suppressed trap-assisted recombination and accounting for the enhanced *V*oc and FF. Furthermore, analysis of the light-intensity-dependent *J*sc yields an α value closer to unity (Fig. S34), while TPC shows a shorter charge transfer lifetime (4.66 vs. 6.59 μs, Fig. S35, Eqs. S5 and S6), both of which signify enhanced charge collection efficiency. Moreover, owing to the superior quality of the buried HTL/perovskite interface, the target device exhibits reduced leakage current in dark *J–V* measurements (Fig. S36).

We also conducted a systematic assessment of the stability of unencapsulated PSCs under multiple stress conditions. In steady‑power output (SPO) analysis, the target devices maintain a high and stable PCE of 25.84%, substantially outperforming the control devices and demonstrating superior operational stability (Fig. 4e). Under accelerated thermal aging at 85 °C in N_2_ for 500 h, the target devices retain 80.81% of their initial PCE, demonstrating superior thermal stability relative to the control devices (61.60% retention, Fig. 4f). Additionally, the fluorine-containing 3F-2TC increases the hydrophobicity of the resultant perovskite surface, as indicated by an increased water contact angle (Fig. S37), thereby contributing to enhanced moisture resistance. Long-term stability was examined under continuous white LED illumination (100 mW cm^−2^, 35% relative humidity). Impressively, the target devices maintained 90.43% of their initial PCE even after 1600 h of continuous operation, representing a substantial improvement over the control devices, which retained only 71.69% of their initial PCE (Fig. 4g). The remarkable long-term stability mainly originates from the combined effects of increased surface hydrophobicity, improved perovskite film quality with less defects, and optimized properties of the buried HTL/perovskite interface.

## Conclusion

In summary, this work establishes the critical role of the buried HTL/perovskite interface in governing lattice strain, a relationship decisively revealed through harsh reverse-bias stress as a diagnostic tool. To this end, we introduce the buried interface engineering strategy based on incorporating 3F-2TC to directly tune the perovskite lattice strain. This ‘strain-first’ approach goes beyond conventional defect passivation by addressing the root cause of instability. Our results demonstrate that 3F-2TC incorporation facilitates the formation of a uniform HTL with improved conductivity and favorable energy-level alignment. Simultaneously, 3F-2TC provides electronegative moieties (F and C=O) that passivate perovskite defects and act as a crystallization template, steering perovskite growth toward reduced lattice strain and enabling efficient charge transport. As a result, the resulting devices achieve a notable PCE of 26.10% with a high *V*oc of 1.19 V. Furthermore, the devices exhibit markedly enhanced stability under multiple stress conditions: reverse bias of − 1.0 V (*T*_90_ lifetime of ~ 200 h), thermal stress at 85 ℃ (*T*_80_ lifetime of ~ 500 h), and continuous illumination with 1-sun LED light (*T*_90_ lifetime of ~ 1600 h). This work underscores that buried interface engineering for lattice strain modulation provides a powerful strategy to boost the efficiency and stability of PSCs.

## Supplementary Information

Below is the link to the electronic supplementary material.

Supplementary file1 (DOCX 5976 KB)
