# Supplementary material for: Taming Lattice Strain via Buried Interface Engineering for Reverse-Bias Resilient Perovskite Solar Cells
Source: Nanomicro Lett. 2026 May 27;18:387. doi: 10.1007/s40820-026-02244-2 (PMC13212845; doi:10.1007/s40820-026-02244-2)
Supplement: Supplementary file 1 — Supplementary file1 (DOCX 5976 KB) [file 40820_2026_2244_MOESM1_ESM.docx]

Supporting Information for

**Taming Lattice Strain via Buried Interface Engineering for Reverse-Bias Resilient Perovskite Solar Cells**

Niqian Du^1,2^†, Shanshan Du^1,2^†, Yaru Du^1^†, Xiaobo Zhang^2^*, Xiaoyi Hou^2^, Chi Feng^1^, Rongdong Xiang^1^, Xin Wu^3^, Heping Fu^1^, Zhiyong Liu^1^*, Tingwei He^4^, Kaikai Liu^2^*

^1^ Henan Key Laboratory of Advanced Semiconductor & Functional Device Integration, School of Physic, Henan Normal University, Xinxiang 45007, P. R. China

^2^ Henan Key Laboratory of Optoelectronic Energy Storage Material and Application, School of Physics and Engineering, Henan University of Science and Technology, Luoyang 471023, P. R. China

^3^ Department of Chemistry, City University of Hong Kong, Kowloon 999077, Hong Kong, P. R. China

^4^ College of Physics Science and Technology, Hebei University, Baoding 071002, P. R. China

† Niqian Du, Shanshan Du, and Yaru Du contributed equally to this work.

* Corresponding authors. E-mail: [xiaobo@haust.edu.cn](mailto:xiaobo@haust.edu.cn) (Xiaobo Zhang); [zyliu01@163.com](mailto:zyliu01@163.com) (Zhiyong Liu); [kkliu@haust.edu.cn](mailto:kkliu@haust.edu.cn) (Kaikai Liu)

**S1 Supplementary Equations and Tables**

**Equation S1**

The following formula was used for SCLC analysis of pure hole devices (ITO/Mixed or Control SAMs/PVK/PTAA/Ag), and the defect density Nt was obtained.

$\begin{aligned} N_{t}=\frac{2\varepsilon_{r}\varepsilon_{0}V_{TFL}}{eL^{2}} \end{aligned}$ (S1)

Where $\varepsilon_{r}$, $\varepsilon_{0}$ and $L$ represent the relative perovskite dielectric constant, vacuum dielectric constant and film thickness, respectively, and e is the basic charge. $V_{TFL}$ was obtained by tangential method with readouts of 0.66 V and 0.83 V respectively.

**Equation S2**

The formula for calculating electrical conductivity (σ)

$\begin{aligned} \sigma\mathbf{=}\frac{d}{AR} \end{aligned}$ (S2)

Where $d$, $A$ and $R$ are respectively the thickness, area and resistance of the hole layer.

**Equation S3**

The Mott-Schottky equation

$\begin{aligned} \frac{1}{c^{2}}=\frac{2(V_{bi}-V)}{A^{2}e\epsilon\epsilon_{0}N_{A}} \end{aligned}$ (S3)

Where, $A$ is the active area, $c$ is the device capacitance, $V$ is the applied bias, and $N_{A}$ is the doping density.

**Equation S4**

Hysteresis calculation formula for perovskite solar cells

$HI=\frac{{PCE}_{reverse}-{PCE}_{forward}}{{PCE}_{reverse}}$ (S4)

**Equation S5 and S6**

The fitting formula and the calculation formula for the average carrier lifetime of TPC/TPV

$\begin{aligned} f\left( t \right)=A_{1}\exp\left( -\frac{t}{\tau_{1}} \right)+A_{2}\exp\left( -\frac{t}{\tau_{2}} \right) \end{aligned}$ (S5)

$\begin{aligned} \tau_{ave}=\frac{A_{1}\tau_{1}^{2}+A_{2}\tau_{2}^{2}}{A_{1}\tau_{1}+A_{2}\tau_{2}} \end{aligned}$ (S6)

Where $A_{1}$ and $A_{2}$ are attenuation amplitudes,$\tau_{1}$and $\tau_{2}$ are attenuation times,$\tau_{ave}$ average decay time.

**Equation S7**

The calculation formula of illumination ideal factor (n)

$\begin{aligned} n_{id}=\frac{q}{kT}\times\frac{dV_{OC}}{dln\left( L \right)} \end{aligned}$ (S7)

Where $L$ is the normalized light intensity, $k$ is the Boltzmann constant, $T$ is the absolute temperature, and $q$ is the unit charge. The closer the $n$ value is to 1, the lower the carrier recombination caused by the defect.

**Table S1** TRPL parameters of the Control and Target SAM films

| Sample | A_1_ (%) | τ_1_ (ns) | A_2_ (%) | τ_2_ (ns) | Τ_ave_ (ns) |
| --- | --- | --- | --- | --- | --- |
| Control | 58.01 | 113.79 | 41.99 | 564.54 | 466.37 |
| Target | 44.33 | 193.52 | 55.67 | 743.05 | 648.66 |

**Table S2** Fitting parameters of the normalized GSB decays at 766 nm for the perovskite films deposited on the HTL/ITO substrate without and with 3F-2TC modification

| Sample | A_1_ (%) | τ_1_ (ps) | A_2_ (%) | τ_2_ (ps) | Τ_ave_ (ns) |
| --- | --- | --- | --- | --- | --- |
| Control | 40.80 | 263.75 | 59.20 | 1842.13 | 1.70 |
| Target | 41.19 | 177.57 | 58.81 | 1717.62 | 1.61 |

**Table S3** EIS fitting parameters of the control and target devices

| Sample | Rs (Ω) | Rrec (Ω) |
| --- | --- | --- |
| Control | 1.47 | 963.05 |
| Target | 0.78 | 1670.69 |

**S2 Supplementary Figures**


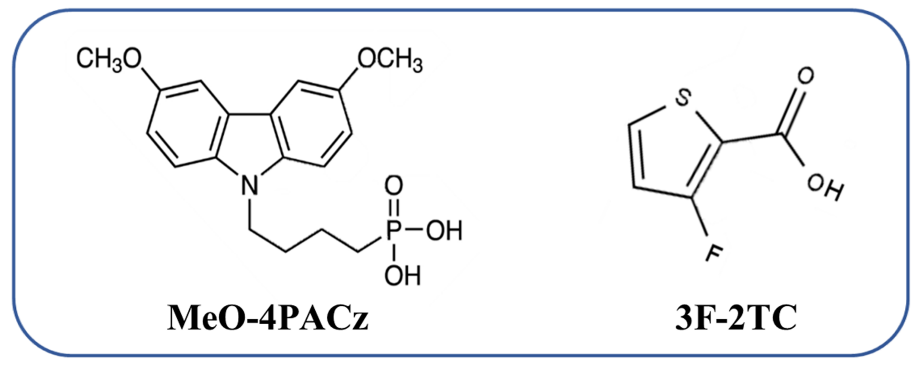


**Fig. S1** The molecular structures of MeO-4PACz and 3F-2TC.

**Fig. S2** *J-V* curves of devices with different volume ratios of MeO-4PACz to 3F-2TC.


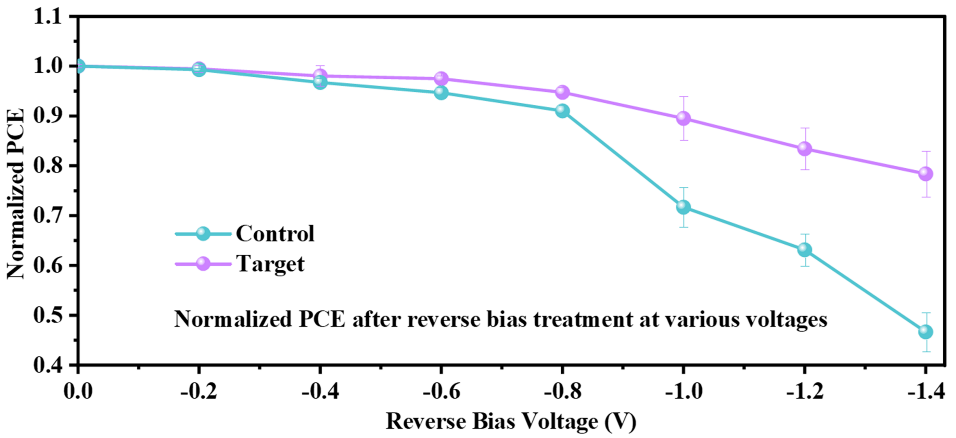


**Fig. S3** The PCE changes of control devices and target divices under different reverse bias**.**


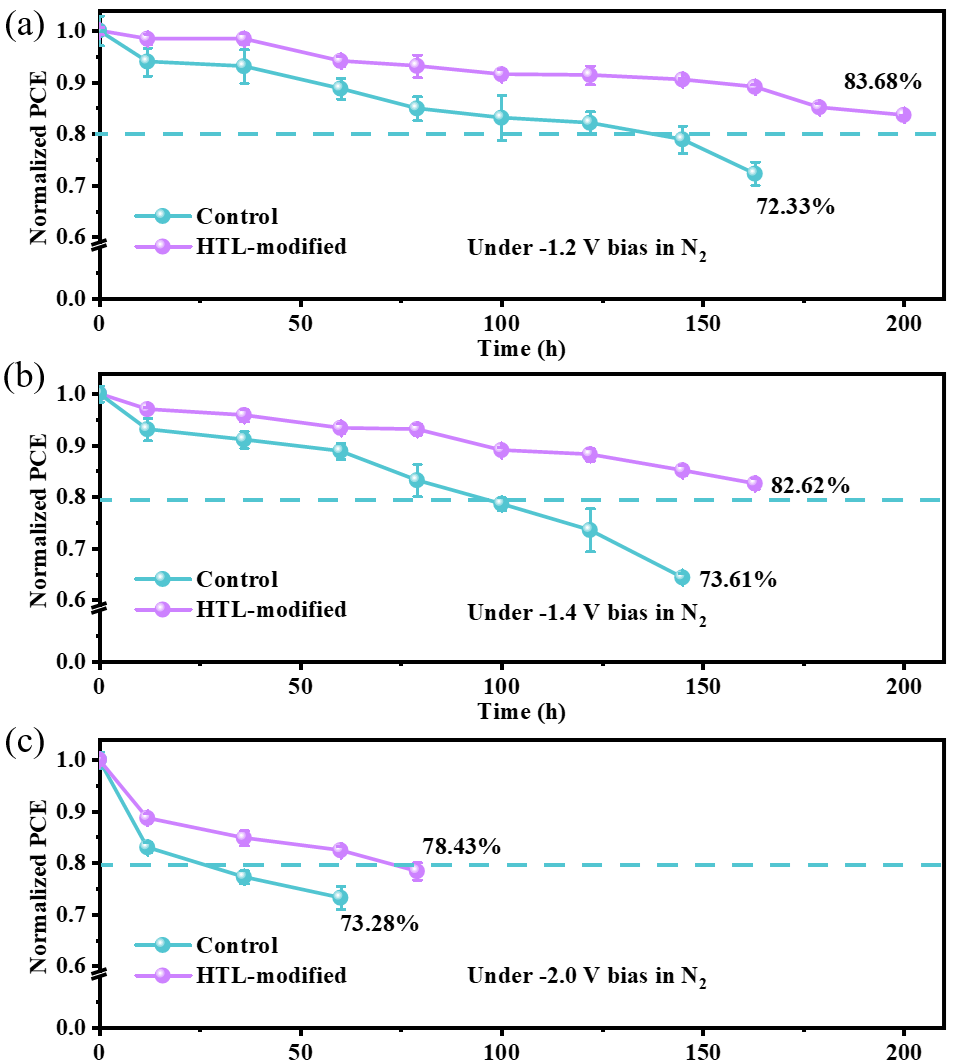


**Fig. S4** Changes in PCE of control and HTL-modified devices under high magnitudes of bias stress for (a) -1.2 V, (b) -1.4 V, and (c) -2.0 V.


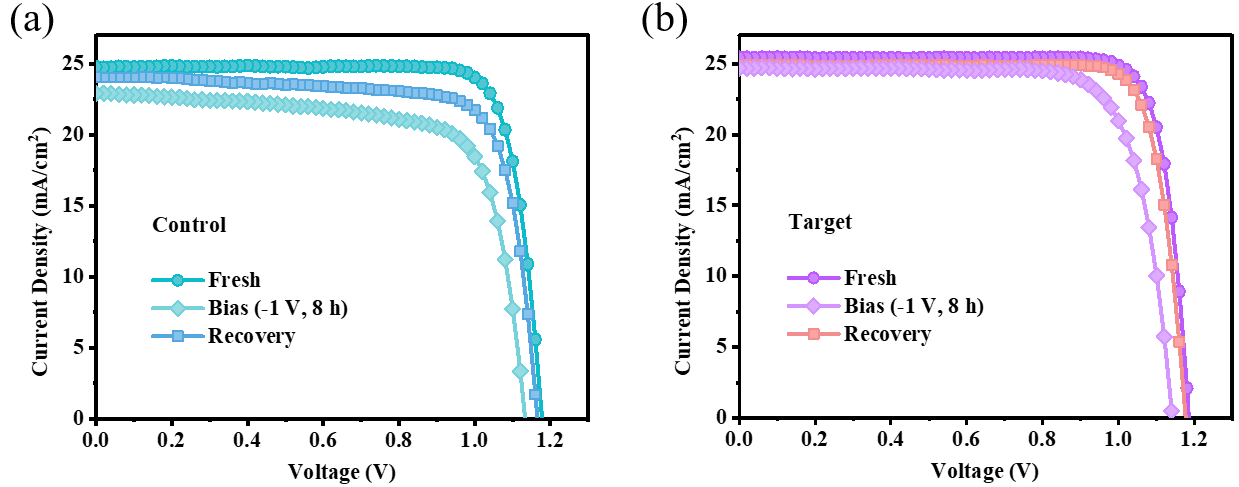


**Fig. S5** *J-V* curves of (a) the control device and (b) the target device under different conditions of the fresh, bias (under -1 V for 8 h), and recovery (overnight dark storage).


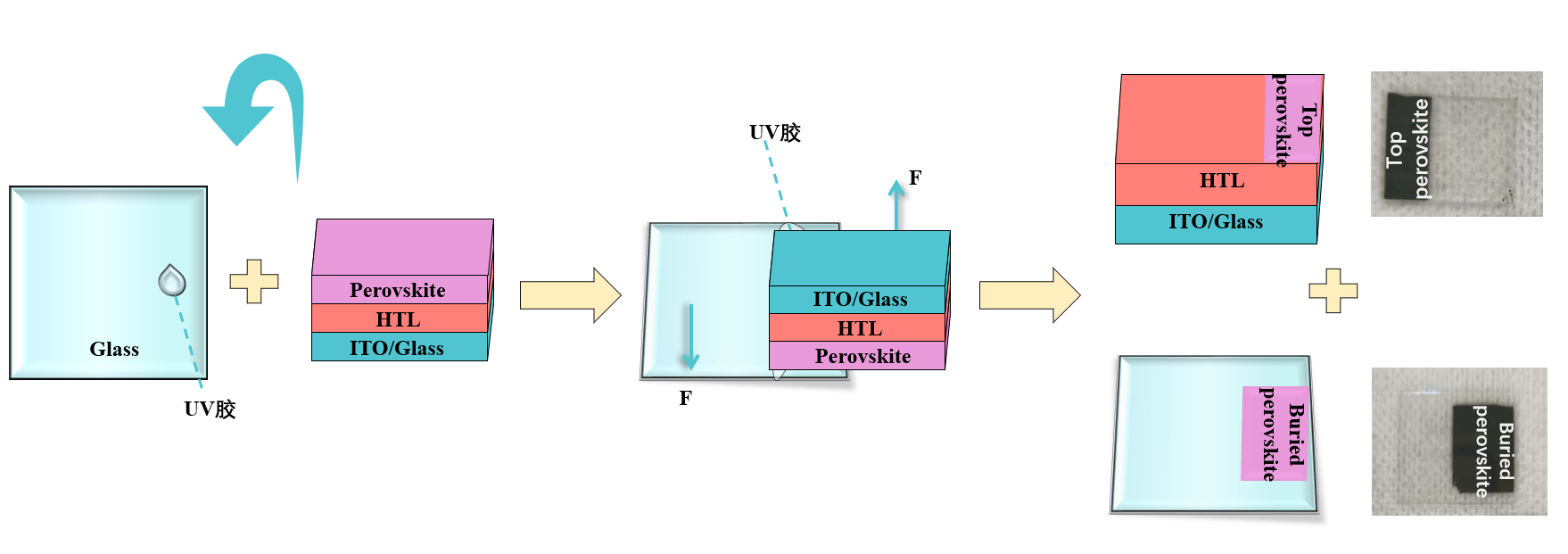


**Fig. S6** Schematic illustration of preparation process to expose the buried interface.

**Fig. S7** Temperature-dependent conductivity of the control and target device.

**
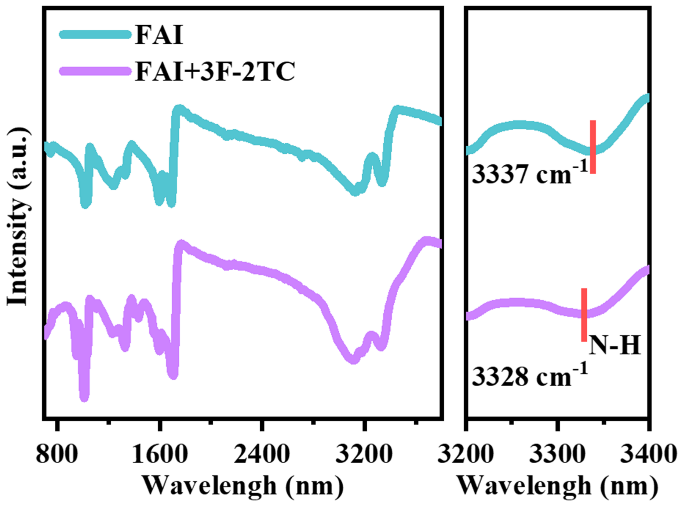
**

**Fig. S8** FTIR spectra of FAI and FAI/3F-2TC mixture.

**
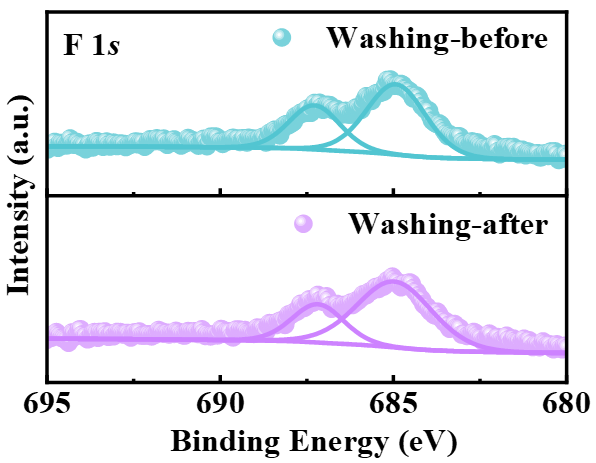
**

**Fig. S9** F 1s signal in XPS spectra of the target HTL films before and after DMF/DMSO washing.


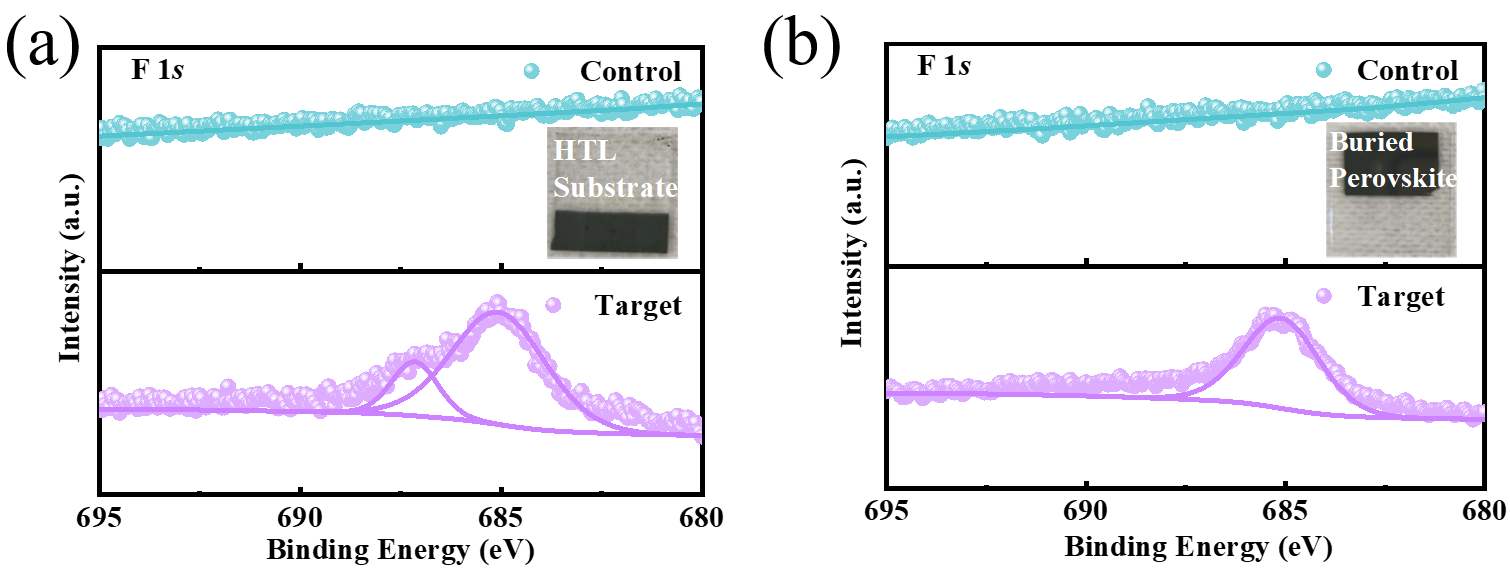


**Fig. S10** F 1s signal in XPS spectra of (a) the HTL substrate (b) the buried perovskite films.

**
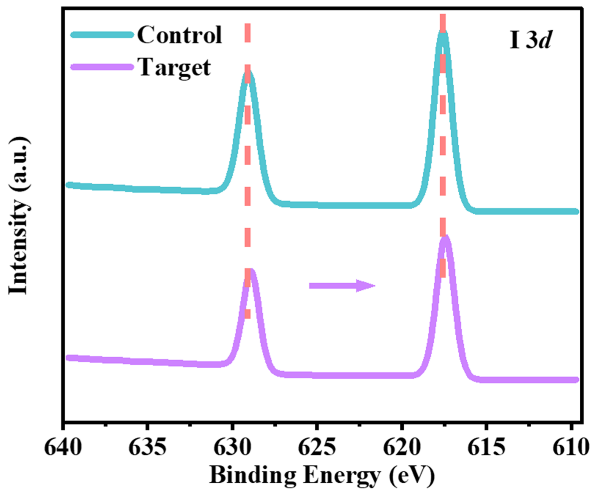
**

**Fig. S11** I 3*d* signal in XPS spectra for the buried interface of the control and target perovskite films.


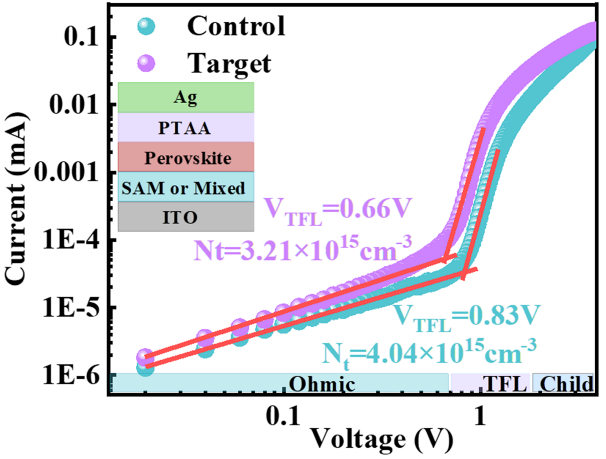


**Fig. S12** SCLC analysis of hole-only devices based on the control and target perovskite film.


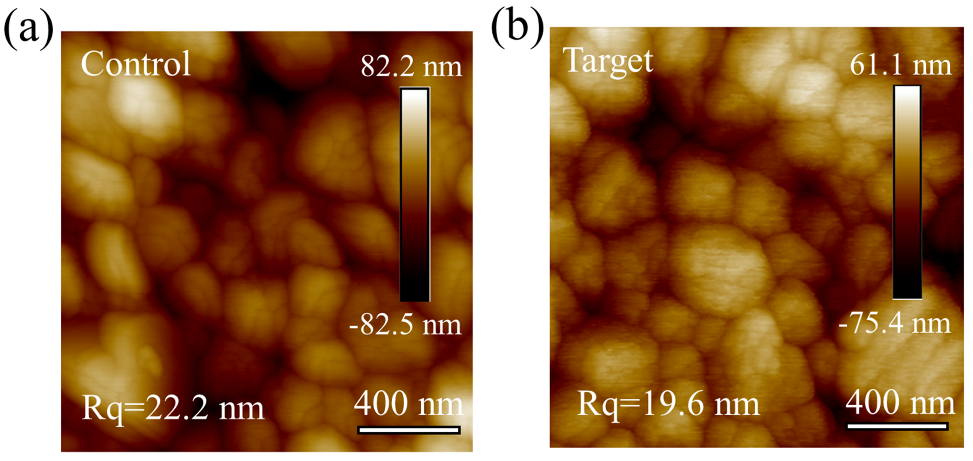


**Fig. S13** AFM images of the top surface of the control and target perovskite film.


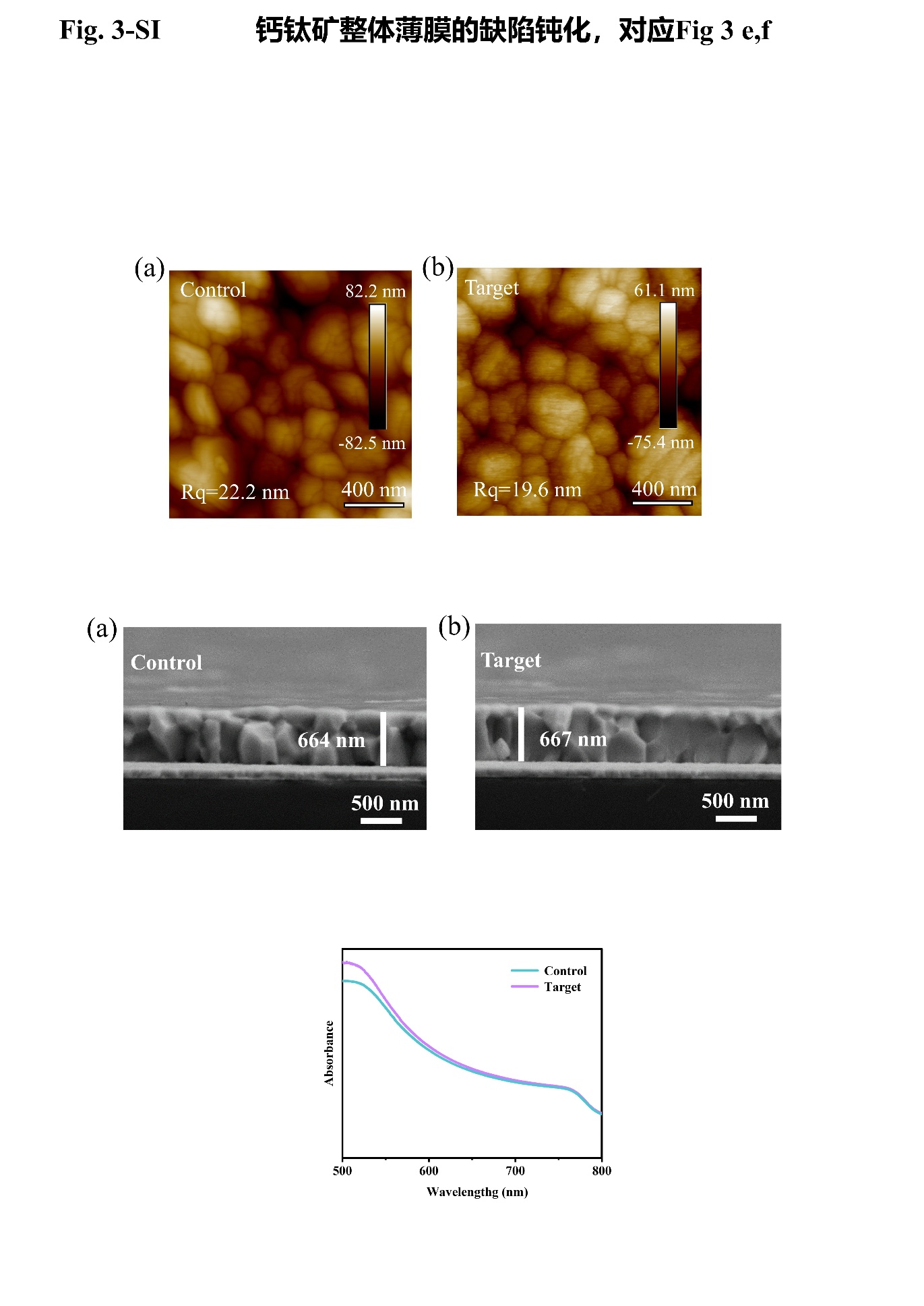


**Fig. S14** Cross-sectional SEM images of the control and target perovskite film.


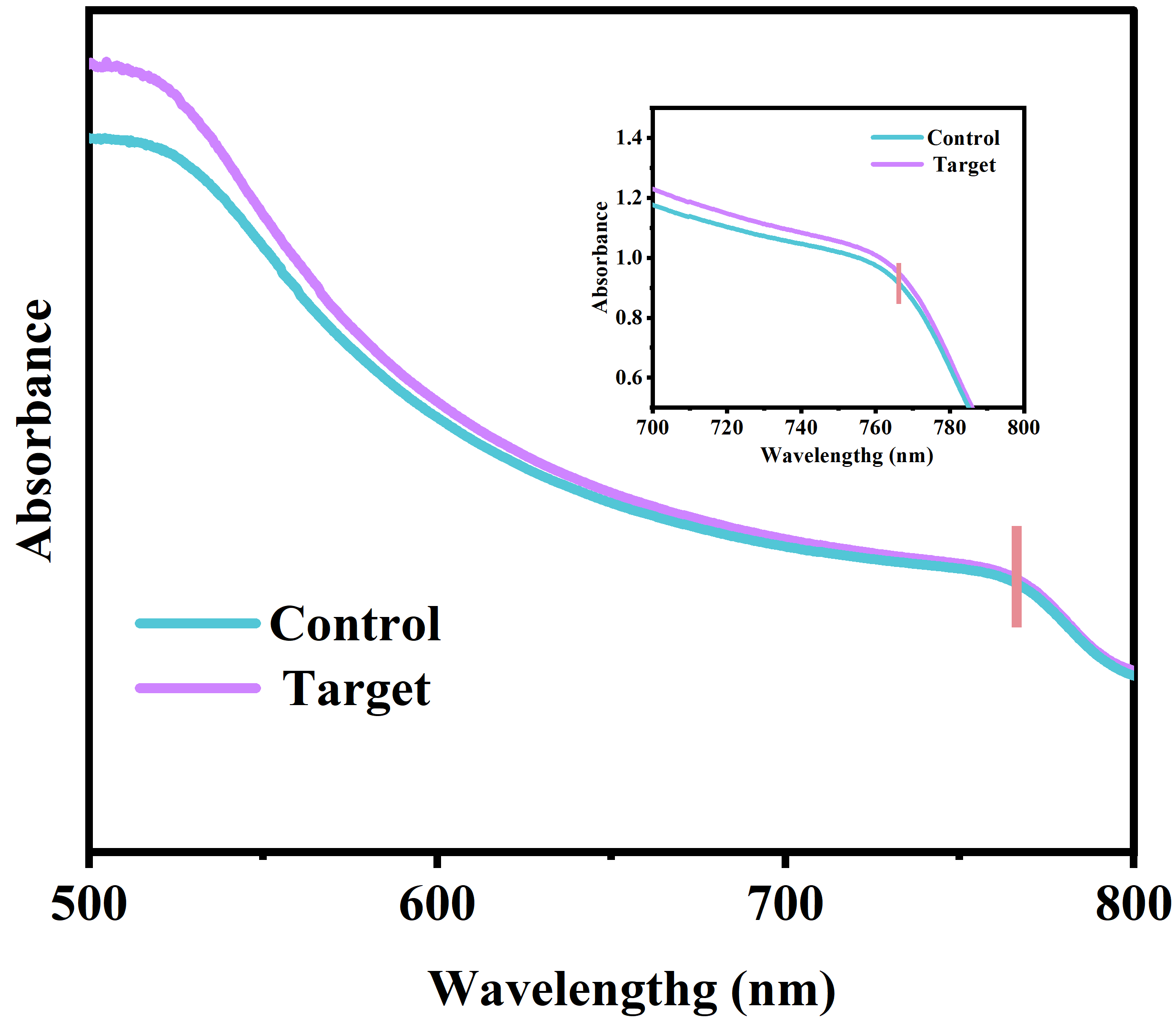


**Fig. S15** Absorption of the control and target perovskite film.

**
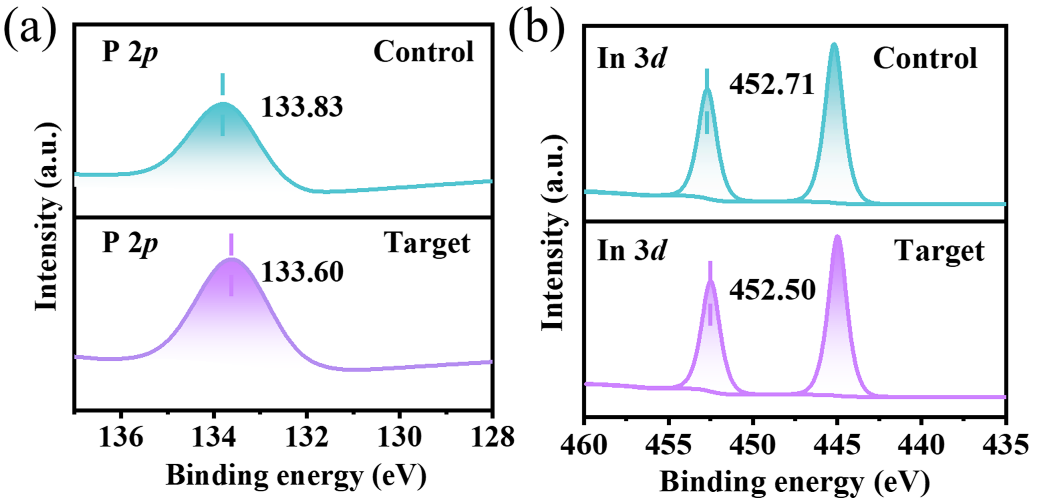
**

**Fig. S16** (a) P 2*p* and (b) In 3*d* in XPS spectra for the control HTL and target HTL.


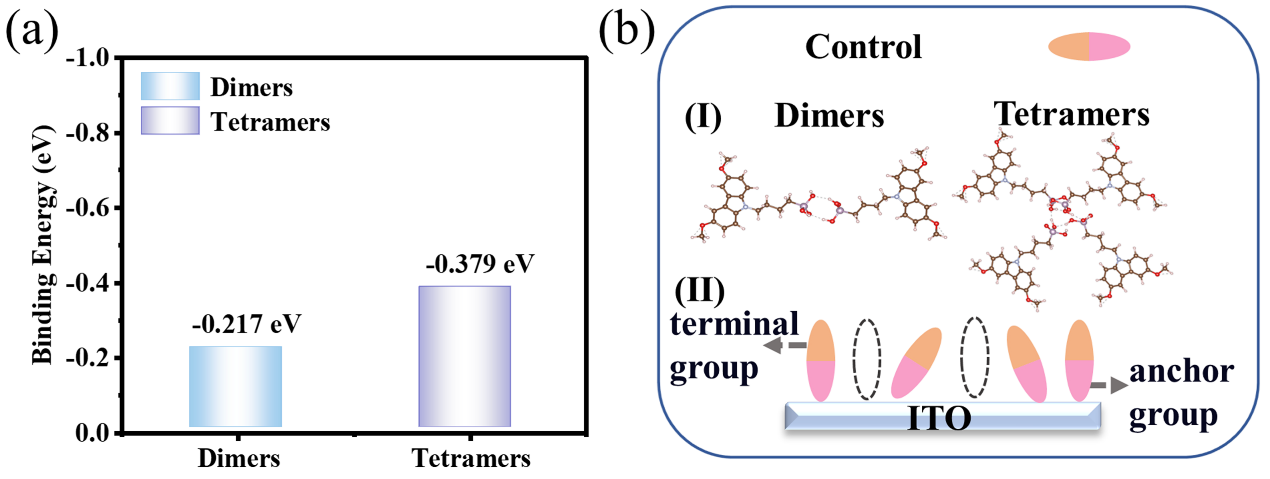


**Fig. S17** (a) Binding energy of the dimers and tetramers for MeO-4PACz molecules. (b) DFT-simulated conformations of MeO-4PACz dimers and tetramers (part Ι) and diagram of the control HTL (part Ⅱ).

**Fig. S18** DLS measurements of the MeO-4PACz solutions without (control) and with (Target) 3F-2TC incorporation.


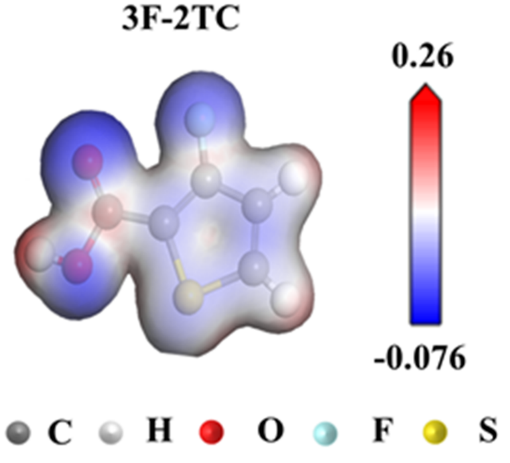


**Fig. S19** Electrostatic surface potential (ESP) maps of the 3F-2TC molecule.


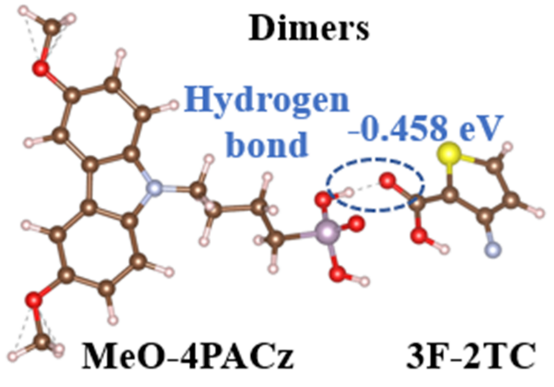


**Fig. S20** DFT-simulated binding energy of the MeO-4PACz and 3F-2TC heterodimer.


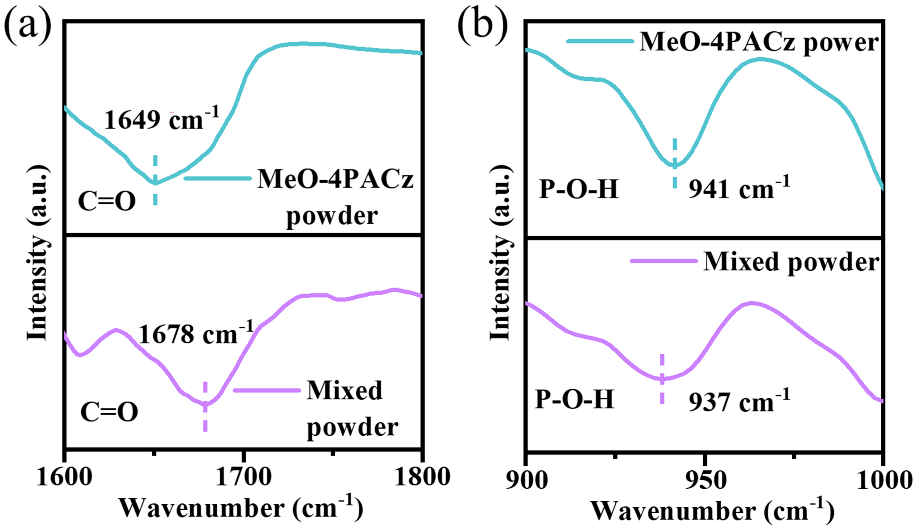


**Fig. S21** FTIR spectra of the MeO-4PACz powder and MeO-4PACz/3F-2TC mixed powder.

**
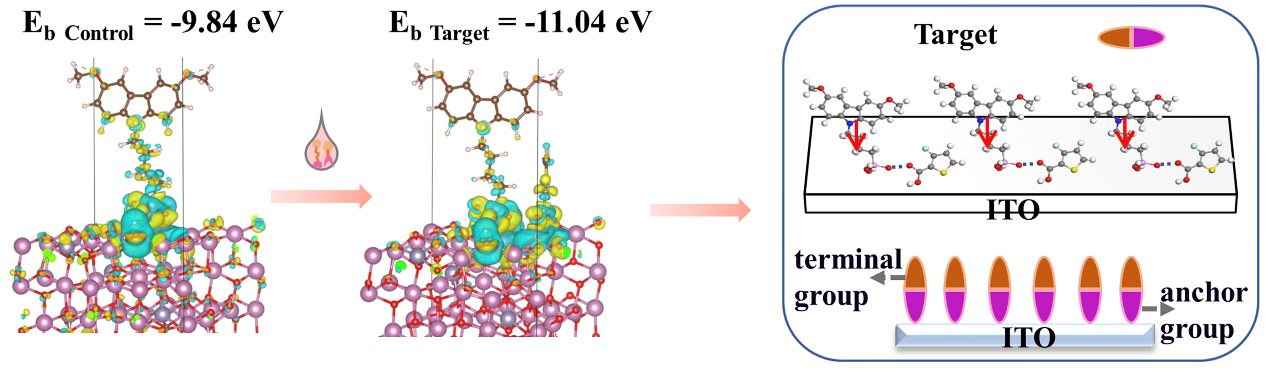
**

**Fig. S22** Mechanism diagram and schematic illustration of depositing the target HTL (MeO-4PACz/3F-2TC) on ITO substrates.


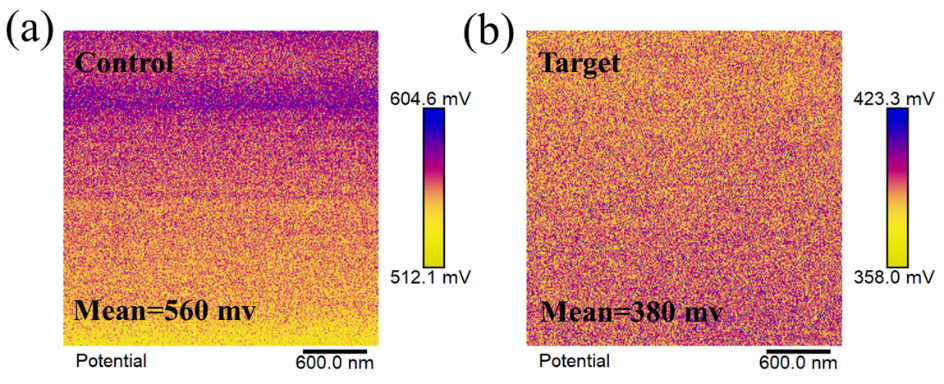


**Fig. S23** KPFM images of the control HTL and target HTL.


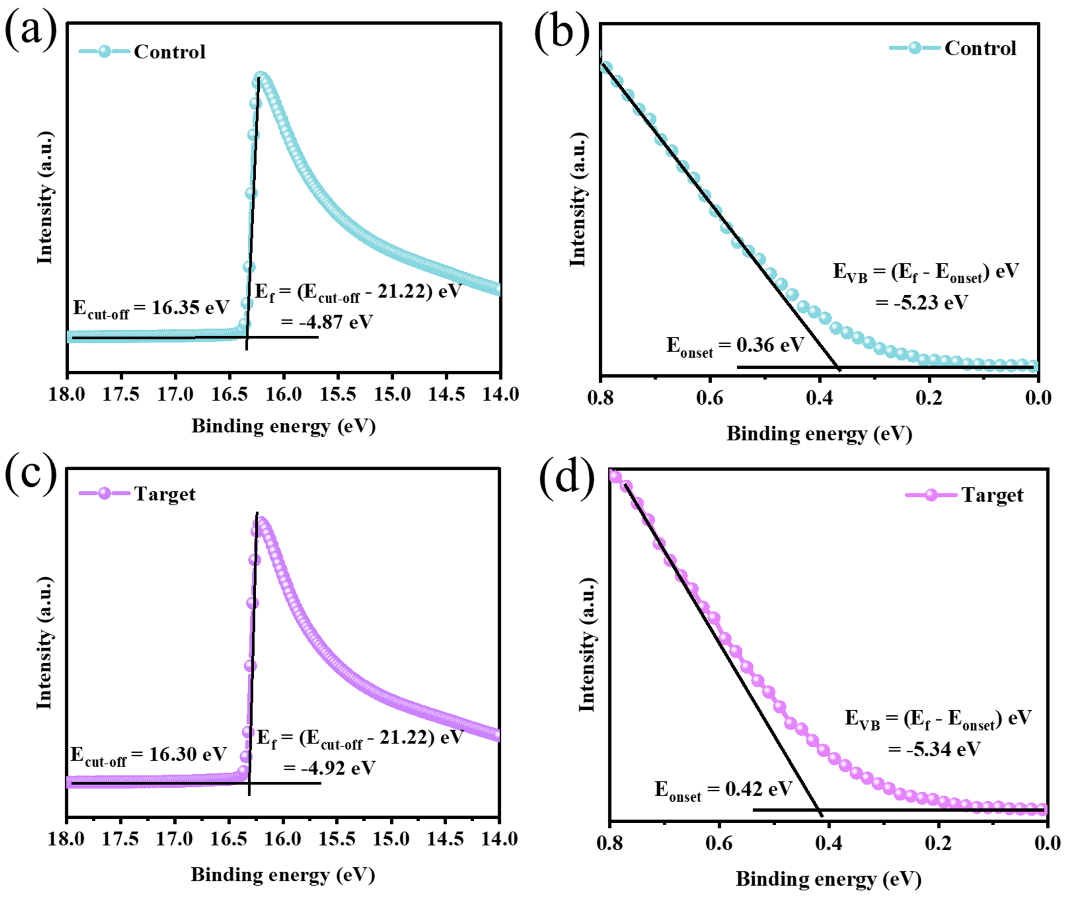


**Fig. S24** UPS spectra for the (a, b) control HTL and (c, d) target HTL.


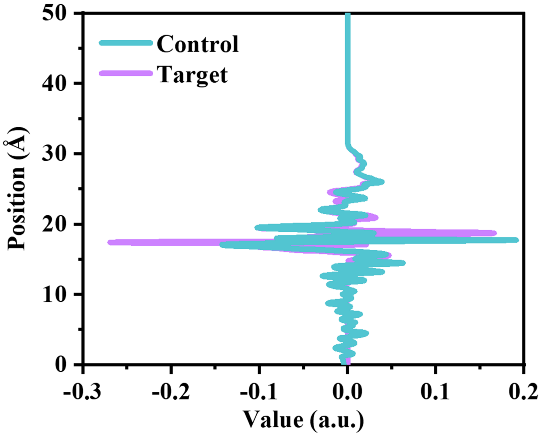


**Fig. S25** Differential charge density of the interface for the control HTL and target HTL.


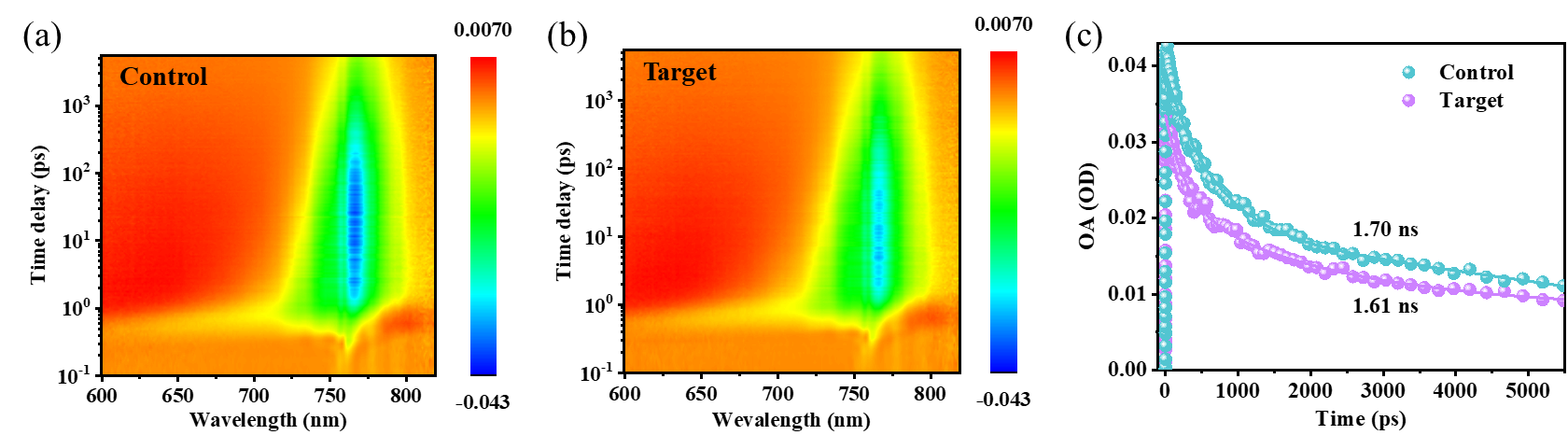


**Fig.** **S26** (a, b) 2D pseudo-color plots of fs-TAS for the perovskite film deposited on HTL/ITO substrate without (control) and with (target) 3F-2TC modification; (c) Kinetic traces of the ground state bleaching (GSB) signal probed at 766 nm, normalized by the steady-state absorbance at 766 nm.


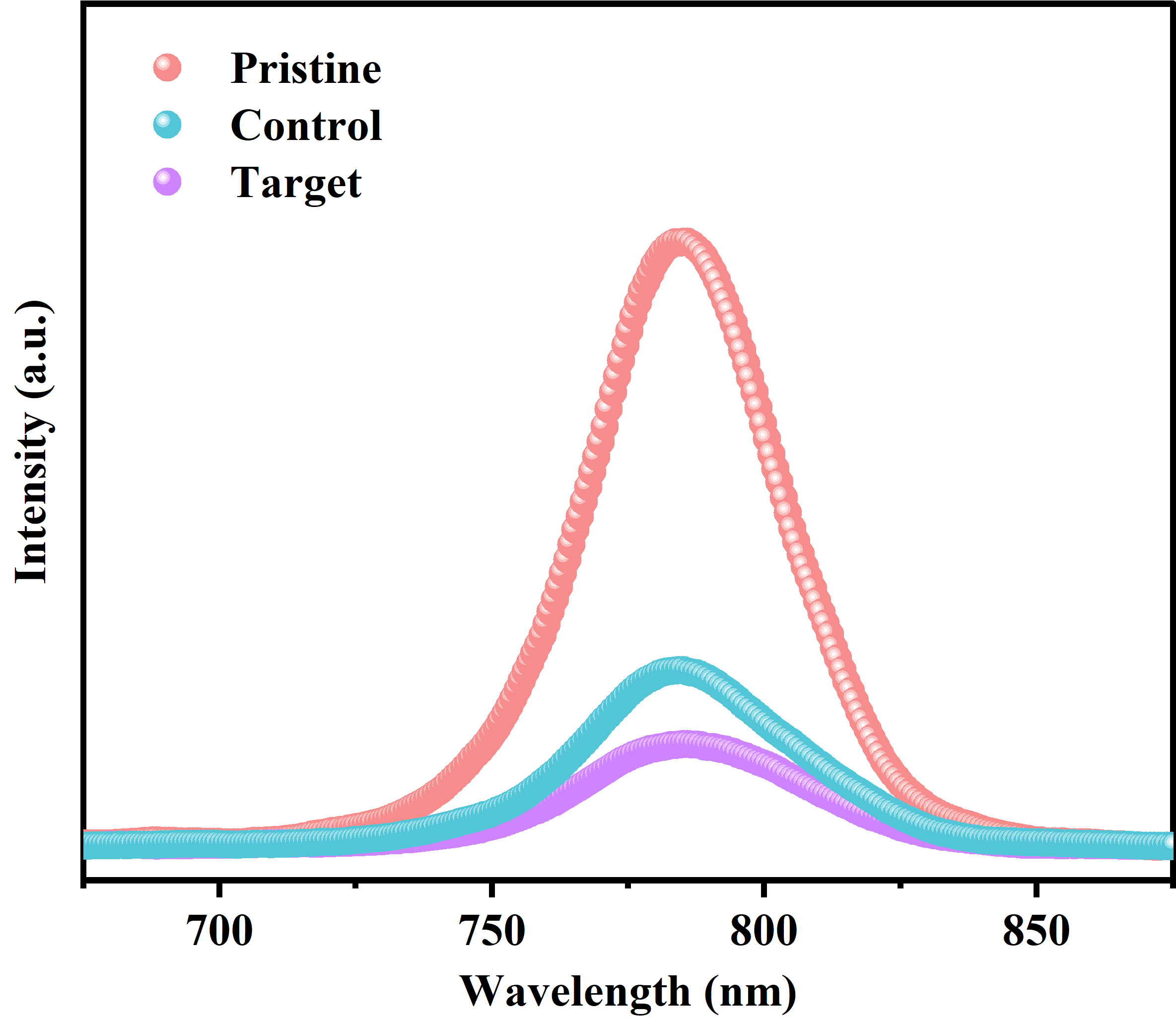


**Fig.** **S27** Steady-state PL spectra of Glass/PVK (Pristine), Glass/ITO/MeO-4PACz/PVK (Control), and Glass/ITO/MeO-4PACz+3F-2TC/PVK (Target).


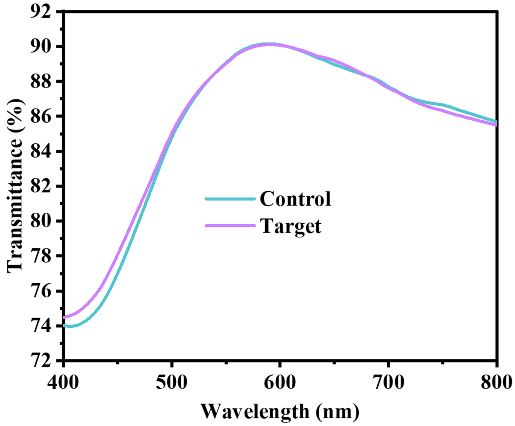


**Fig. S28** Transmission spectra of the control HTL and target HTL.


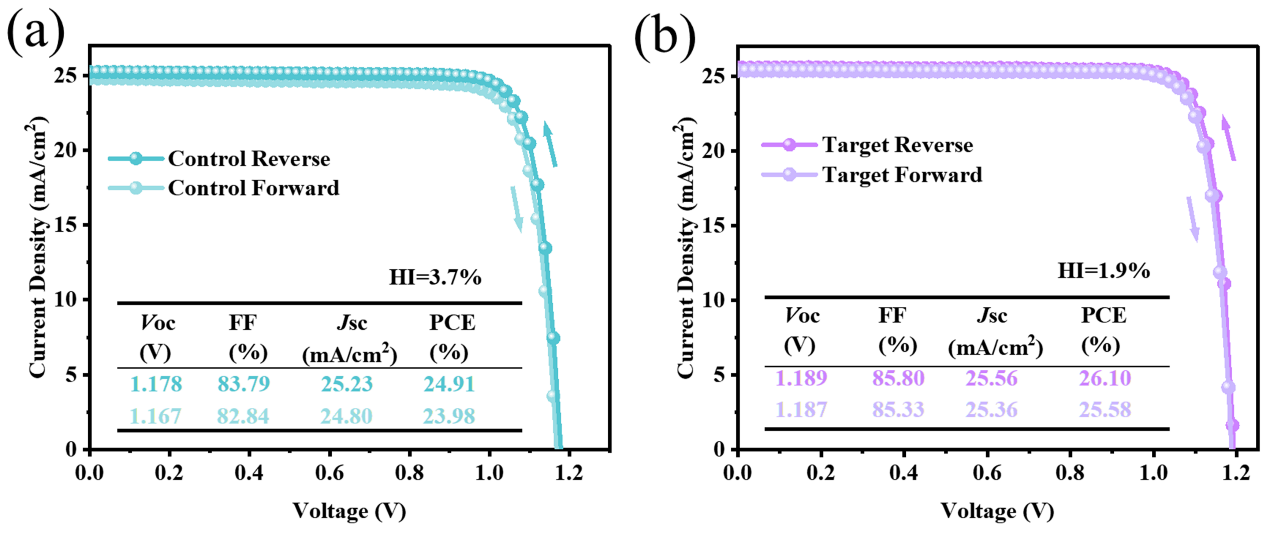


**Fig. S29** *J-V* curves of the control and target devices under the reverse and forward scanning modes.


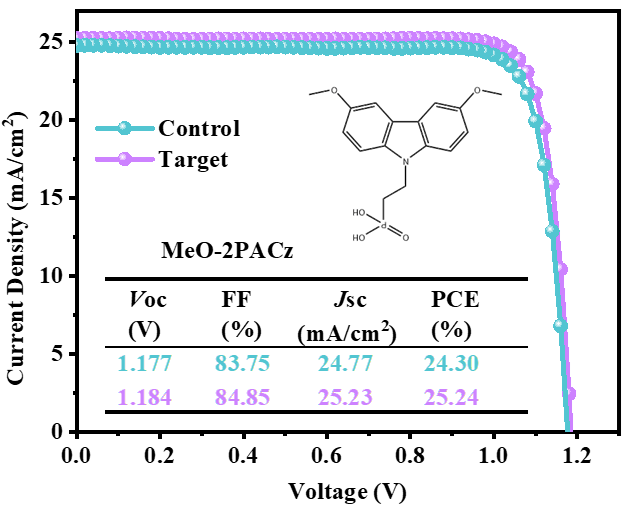


**Fig. S30** *J-V* curves of the best-performing PSCs based on MeO-2PACzHTLs without (control) and with (target) 3F-2TC modifiction.


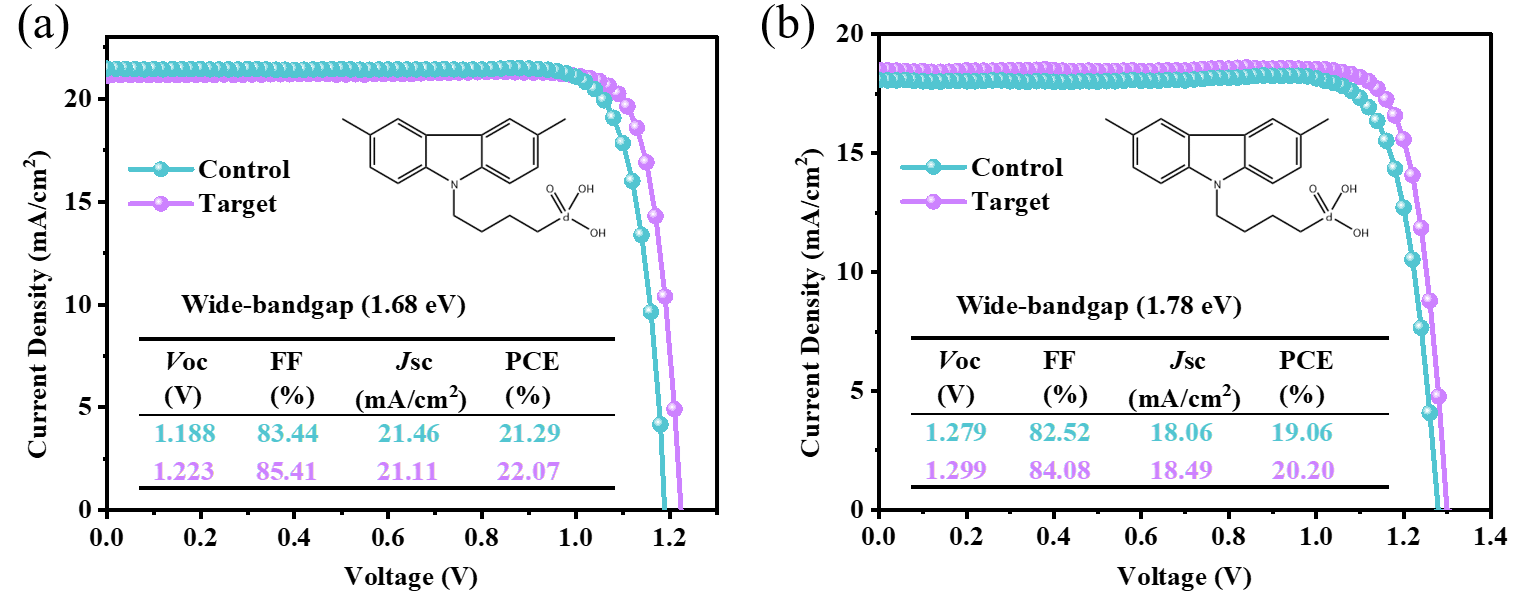


**Fig. S31** *J-V* curves of the champion PSCs with different wide-band gaps based on Me-4PACz HTLs without (control) and with (target) 3F-2TC modification.


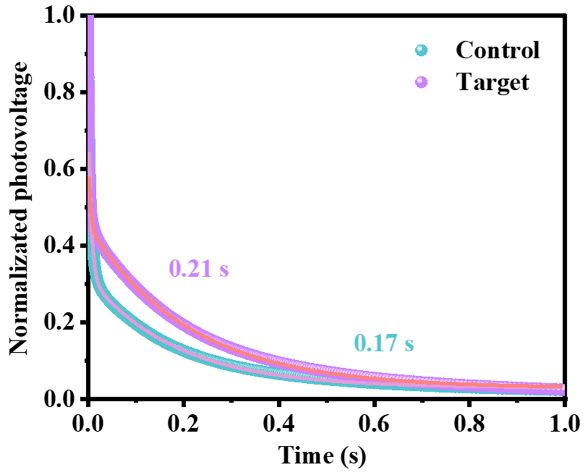


**Fig. S32** TPV decay curves of the control and target device.


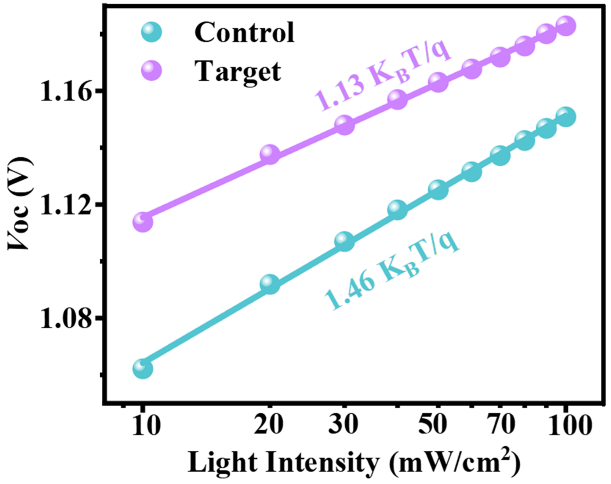


**Fig. S33***V*oc dependence on light intensities for the control and target device.

**
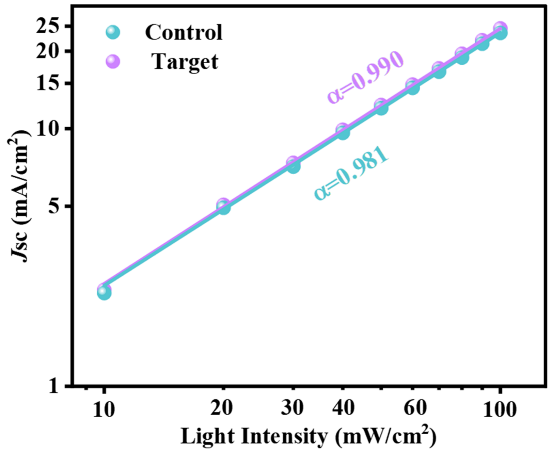
**

**Fig. S34** *J*sc dependence of on light intensities for the control and targe device.


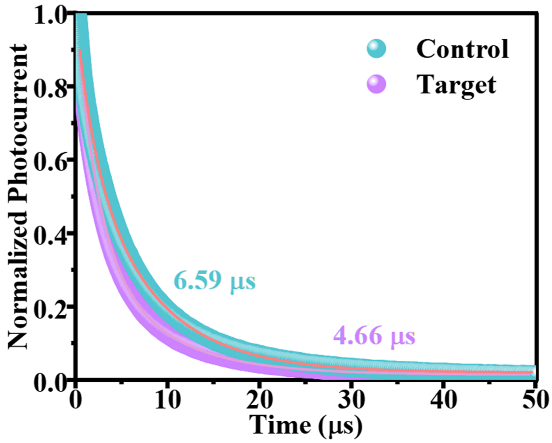


**Fig. S35** TPC decay curves of the control and target device.

**
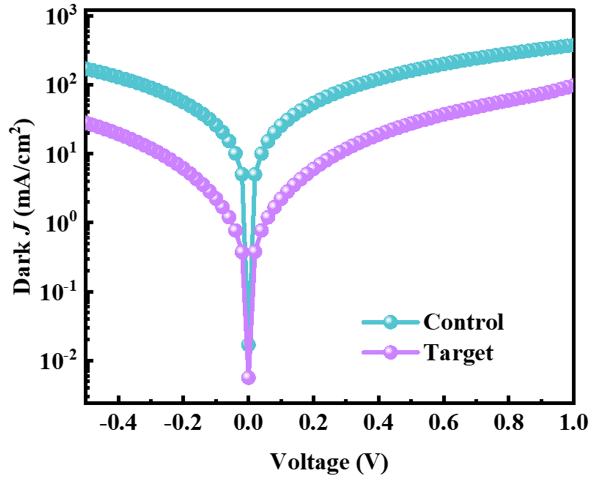
**

**Fig. S36** Dark *J-V* curves for the control and target device.


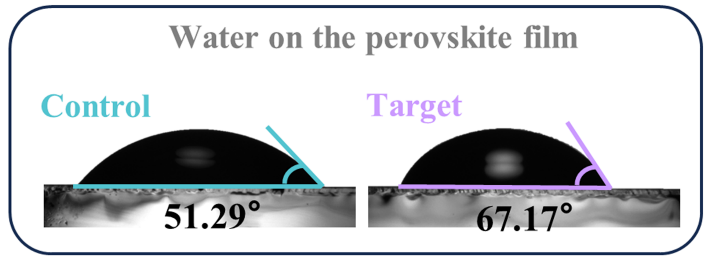


**Fig. S37** Water contact angle of the perovskite films deposited on the control HTL and target HTL.
